# Deep learning for automated scoring of immunohistochemically stained tumour tissue sections – Validation across tumour types based on patient outcomes

**DOI:** 10.1016/j.heliyon.2024.e32529

**Published:** 2024-06-13

**Authors:** Wanja Kildal, Karolina Cyll, Joakim Kalsnes, Rakibul Islam, Frida M. Julbø, Manohar Pradhan, Elin Ersvær, Neil Shepherd, Ljiljana Vlatkovic, OSBREAC, Xavier Tekpli, Øystein Garred, Gunnar B. Kristensen, Hanne A. Askautrud, Tarjei S. Hveem, Håvard E. Danielsen, OSBREAC - Oslo Breast Cancer Consortium, Tone F. Bathen, Elin Borgen, Anne-Lise Børresen-Dale, Olav Engebråten, Britt Fritzman, Olaf Johan Hartman-Johnsen, Øystein Garred, Jürgen Geisler, Gry Aarum Geitvik, Solveig Hofvind, Rolf Kåresen, Anita Langerød, Ole Christian Lingjærde, Gunhild M. Mælandsmo, Bjørn Naume, Hege G. Russnes, Kristine Kleivi Sahlberg, Torill Sauer, Helle Kristine Skjerven, Ellen Schlichting, Therese Sørlie

**Affiliations:** a Institute for Cancer Genetics and Informatics, Oslo University Hospital, NO-0424, Oslo, Norway; b Gloucestershire Cellular Pathology Laboratory, Gloucester, GL53 7AN, UK; c Department of Medical Genetics, Institute of Clinical Medicine, Faculty of Medicine, University of Oslo and Oslo University Hospital, NO-0450, Oslo, Norway; d Department of Pathology, Oslo University Hospital, NO-0424, Oslo, Norway; e Nuffield Division of Clinical Laboratory Sciences, University of Oxford, Oxford, OX3 9DU, UK

**Keywords:** Deep learning, Digital image analysis, Immunohistochemistry, Cancer, Prognosis

## Abstract

We aimed to develop deep learning (DL) models to detect protein expression in immunohistochemically (IHC) stained tissue-sections, and to compare their accuracy and performance with manually scored clinically relevant proteins in common cancer types.

Five cancer patient cohorts (colon, two prostate, breast, and endometrial) were included. We developed separate DL models for scoring IHC-stained tissue-sections with nuclear, cytoplasmic, and membranous staining patterns. For training, we used images with annotations of cells with positive and negative staining from the colon cohort stained for Ki-67 and PMS2 (nuclear model), the prostate cohort 1 stained for PTEN (cytoplasmic model) and β-catenin (membranous model). The nuclear DL model was validated for MSH6 in the colon, MSH6 and PMS2 in the endometrium, Ki-67 and CyclinB1 in prostate, and oestrogen and progesterone receptors in the breast cancer cohorts. The cytoplasmic DL model was validated for PTEN and Mapre2, and the membranous DL model for CD44 and Flotillin1, all in prostate cohorts. When comparing the results of manual and DL scores in the validation sets, using manual scores as the ground truth, we observed an average correct classification rate of 91.5 % (76.9–98.5 %) for the nuclear model, 85.6 % (73.3–96.6 %) for the cytoplasmic model, and 78.4 % (75.5–84.3 %) for the membranous model. In survival analyses, manual and DL scores showed similar prognostic impact, with similar hazard ratios and p-values for all DL models. Our findings demonstrate that DL models offer a promising alternative to manual IHC scoring, providing efficiency and reproducibility across various data sources and markers.

## Highlights

• General deep learning models for the quantification of protein expression in images of immunohistochemically stained tissue sections.
• Separate deep learning models for nuclear, cytoplasmic and membranous protein expression.
• Comparable performance of manual and deep learning-based results in correlations and prognostic analyses.
• The automatic scores were validated in several clinically relevant proteins and multiple cancer types.

## Graphical abstract

**Figure:**
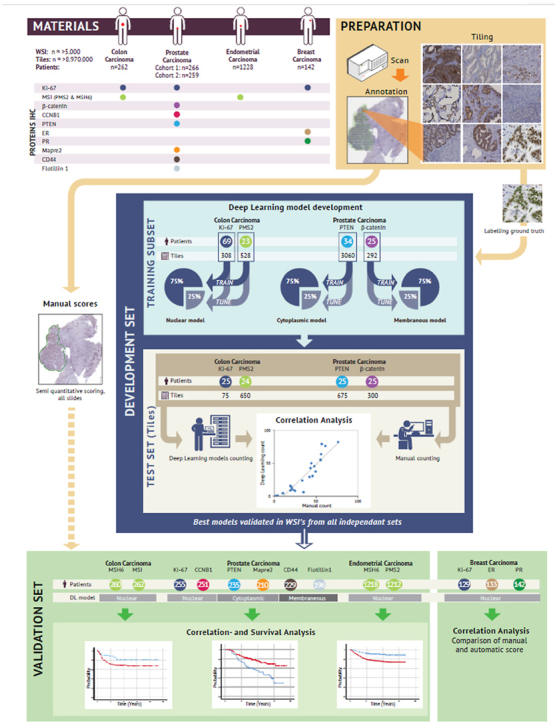

**Table:** 

Glossary	Definition	
*Annotation*	Delineation of the region of interest	
*WSI*	Whole slide image	
*Tile*	The annotated tumour area is divided into small rectangular images, called tiles	
*Ground truth*	Positively and negatively stained tumour cells as indicated by a human expert	
*Labelling*	Manual delineation of positively and negatively stained tumour cells in the development (training and tuning) subset.	
*Manual count*	Using an in-house developed software tool, manual counting involves precise point-annotation of all tumour cells that are positively or negatively stained within a tile. This method provides the exact fraction of positive staining within a tile.	
*Manual scores/Semi-quantitative estimation*	Determining the proportion of positive staining within a WSI using a semi-quantitative approach. This method is less precise compared to manual counting, where each tumour cell is scored.	
*True positive/negative*	An object where the model correctly predicts the ground truth label (positive or negative stain).	
*False positive/negative*	An object where there is disagreement between the model prediction and the ground truth label.	
*Development set*	A collection/set of tiles used for the development of a deep learning model. Each tile is accompanied by its respective ground truth annotations. This set was further classified into training and tuning as given below.	
	*Training subset*	A dataset composed of tiles from approximately two-thirds of the patients from the development set and their corresponding tiles with labelled data. It is used to train the parameters of a deep learning model.
	*Tuning subset*	A dataset composed of tiles from about one-third of the patients from the development set and their corresponding tiles with labelled data. Manual counts are compared to deep learning scores in the tuning subset, and the results are used to select the deep learning models.
*Test set*	*Test set*	A dataset comprising labelled tiles from 25 patients from the development set. It is used to identify the best performing deep learning models by assessing their correlation with manual counts.
	*Development test subset*	A dataset of WSIs with immunohistochemical staining for a specific protein, including cases where a few tiles were used for model training. The set is used for evaluating model performance through survival analyses and comparing results with manual scores.
	*Internal test subset*	A dataset of WSIs with immunohistochemistry staining for a specific protein. None of these images were used for model training. The set is used for evaluating model performance through survival analyses and comparing results with manual scores.
*Validation dataset*	Independent datasets of WSIs from full patient cohorts with immunohistochemistry stains for a specific protein that were not used in the development sets	

## 1 Introduction

Immunohistochemistry (IHC) is an essential part of the diagnostic workup by pathologists. Traditionally, IHC-stained slides are scored by manual counting or semi-quantitative estimation using a microscope. However, such an approach is time-consuming and hampered by inter- and intra-observer variation [[1], [2], [3]]. An accurate and reproducible result is crucial since the pathologists’ interpretation defines diagnosis and guides treatment decisions.

Digital image analysis may allow automated IHC scoring of tissue sections, outperforming manual scoring [4]. The potential of digital pathology has been known for more than four decades [5,6], but, until recently, its use has been hampered by the limited digitalization of routine pathology slides [7,8]. The escalating integration of digitalization within pathology laboratories, generating high resolution whole slide images (WSIs, see glossary), heightens the relevance of digital image analysis in pathology.

Machine learning (ML) refers to a set of algorithms that allow computers to identify the relevant features for a given prediction task. Deep learning (DL) is a ML approach that applies artificial neural networks to learn features and tasks directly from input data. DL has demonstrated exceptional prediction accuracy in a range of computer vision tasks, including tumour detection [9], Gleason grading [10], scoring of tissue stains [[11], [12], [13]], and determining prognosis [14]. Implementation of ML-based methods is expected to reduce the pathologists’ workload, but requires generalizable algorithms [[15], [16], [17]].

Colon, prostate, endometrial, and breast cancers collectively account for 27 % of all cancer cases and 18 % of global cancer-related deaths in 2020 [18]. IHC analysis of markers linked to various cellular processes provides prognostic insights for these cancer types. For instance, microsatellite instability (MSI) is a clinically relevant prognostic marker for colorectal cancer [19] and endometrial carcinoma [20]. The presence of MSI is associated with a more favourable prognosis and can be detected through IHC-staining of mismatch repair proteins like PMS2 (PMS1 Homolog 2, Mismatch repair component) and MSH6 (MutS homolog 6)[21]. The loss of expression of these proteins indicates MSI, while sustained expression suggests microsatellite stability (MSS). Hormone receptors, such as oestrogen (ER) and progesterone receptors (PR), serve as clinical markers and guide treatment decisions for patients with breast carcinomas [22]. Ki-67, a marker of cellular proliferation, is a prognostic marker for patients with breast cancers [24], and automated scoring systems could facilitate its clinical integration [3]. Ki-67 has also shown prognostic significance in various cancer types, including colon [26] and prostate [[23], [25]], although not yet recommended for routine clinical use. CCNB1 (Cyclin B) and Mapre2 (Microtubule-associated protein RP/EB family member 2) are involved in regulating cell cycle progression and spindle assembly during mitosis, serving as promising biomarkers in several cancers, including prostate [27,28]. PTEN (phosphatase and tensin homolog), a tumour suppressor, plays a vital role in controlling various cellular processes, such as proliferation, cell growth, DNA repair, and chromosome segregation [29]. It has been linked to patient outcomes in various cancer types including prostate [30]. Additionally, β-catenin (CTNNB1), CD44, and Flotillin1 are critical for cell adhesion and have implications in cancer development by promoting epithelial-mesenchymal transition [[31], [32], [33], [34]]. Loss of their expression has been associated with enhanced tumour cell survival and migration, leading to poorer patient outcomes in multiple cancer types [34,35].

In this study, we developed separate DL models for the detection of nuclear (Ki-67 and PMS2), cytoplasmic (PTEN) and membranous (β-catenin) IHC expression. The results were compared with data obtained by manual counting in an test set using a predefined framework. The models were validated by applying them to WSIs from cancer patient cohorts, including some cancer types not included in training and proteins not specifically trained for. The results were compared with semi-quantitative estimation in correlation and survival analyses. The nuclear model was validated in Ki-67 (breast and prostate), PMS2 and MSH6 (endometrium), ER and PR (breast), and Cyclin B (prostate), the cytoplasmic model was validated in PTEN and Mapre2 (prostate), and the membranous model was validated in CD44 and Flotillin1 (prostate).

## 2 Materials and methods

### 2.1 Cohorts and patients

This study included surgical resection specimens from 262 patients with stage II colon cancer from the Gloucester Colorectal Cancer study (UK) recruited between 1988 and 1996 [36], two prostate cancer cohorts with 266 and 259 patients, respectively (Cohort 1, The Norwegian Radium Hospital 1987–2005; Cohort 2, The Norwegian Radium Hospital, 2001–2006) [30,37], 1228 patients with endometrial cancer (The Norwegian Radium Hospital between 2006 and 2017), and 142 patients with breast cancer from the Oslo 2 (OSL2) study recruited between 2006 and 2016 [38,39]. The studies were approved by the Regional Committees for Medical and Health Research Ethics (REK), Norway (Prostate REK no. S-07443a, Colon REK no 2015/1606, Breast REK no 2006.1607 with amendment 2007.1125, Endometrial REK no 2014/701). Supplementary Fig. 1 describes the flow of patients through the study, including the number of patients included in each stage of the analysis and the reasons for exclusions if applicable. The clinicopathological characteristics are summarised in Supplementary Table 1 for the colon cohort, Supplementary Table 2 for the prostate cohorts, Supplementary Table 3 for the breast cohort, and Supplementary Table 4 for the endometrial cohort.

### 2.2 Immunohistochemistry

A three μm section was cut from each formalin-fixed paraffin-embedded tumour block, mounted on Superfrost plus slides (Thermo Scientific, Waltham, MA), and treated for 1 hour at 60 °C. The EnVision FLEX+ system (Agilent Technologies, Santa Clara, CA) and Dako Autostainer Link 48 (Agilent Technologies) were used for IHC-staining, with antibodies listed in Supplementary Table 5. IHC was performed for Ki-67 in the colon, prostate, and breast cancer sets, for PMS2 and MSH6 in the colon and endometrial cancer set, for PTEN, CCNB1, CD44, Flotillin1, Mapre2 and β-catenin in the prostate cancer sets, and for ER and PR in the breast cancer set (Supplementary Fig. 1 and Supplementary Tables 1–4). Each run included both positive and negative controls. Haematoxylin was used for counterstaining. Breast cancer tissue sections were cut and IHC-stained in the routine clinical laboratory at OUH, while all other sections were prepared and IHC-stained at the Institute for Cancer Genetics and Informatics (ICGI). All sections were scanned at the highest resolution available (termed 40x) by either NanoZoomer XR (Hamamatsu Photonics, Hamamatsu, Japan), NanoZoomer (Hamamatsu Photonics, Hamamatsu, Japan), or Aperio AT2 (Leica Biosystems, IL, US) to yield 15 sets of WSIs, one for each of the studied protein-cancer type combinations. All slides from each set were scanned by the same scanner.

### 2.3 Protein expression and manual scoring

Blinded to clinicopathological- and outcome data, one or two independent observers scored all WSIs for each set by semi-quantitative estimation of the fraction of tumour cells with positive IHC staining (manual scores), (Supplementary Table 6). In total, 6 different human experts were involved in manual scoring of the development test subsets and validation sets. For the detailed description of protein expression and scoring see Supplementary Fig. 2 and Supplementary text. A tumour was categorized as MSI if there was a loss of either PMS2 or MSH6 expression, and as MSS if the expression of both of these proteins was sustained.

### 2.4 Automated scoring by deep learning

The tumour areas were annotated manually by trained personnel using an in-house developed software. Tiles measuring 500μmx500 μm (1024x1024 pixels, 0.488μm/pixel) were generated within the annotated areas. Ki-67-colon, PMS2-colon, PTEN-prostate and β-catenin-prostate sets were divided into two subsets: one for development (including train and tune), and the other for internal test as illustrated in the graphical abstract.

#### 2.4.1 Development of deep learning models

Individual DL models were trained for nuclear, cytoplasmic, and membranous IHC staining patterns, described in the following paragraphs.

The development set for the nuclear model consisted of 308 tiles (800x800 pixels) from 69 WSIs from the Ki-67-colon set and 528 tiles (800x800 pixels) from 23 WSIs from the PMS2-colon set (Supplementary Table 7). The WSIs were tiled in full (40x) resolution. Contours of all identified tumour nuclei were manually annotated and labelled as Ki-67-positive or Ki-67-negative in the 308 tiles (38957 nuclei) from the Ki-67-colon set using in-house developed software. These tiles were used as the ground truth in the training and tuning of the model and were randomly split into training (75 %) and tuning (25 %) at the patient level such that all images from a patient were put in either the training or the tuning partition. The Mask R–CNN–network [53] was trained to segment cells and classify nuclei as Ki-67 positive or Ki-67 negative. The initial model trained using 308 tiles from the Ki-67-colon set was applied to the 528 tiles from the PMS2-colon set to obtain labelled cell nuclei with a predicted PMS2 status and the labelling of nuclei was then manually corrected when required, resulting in 60216 labelled nuclei. A third class, internal positive controls (non-tumour cells expressing PMS2) were added (6294 nuclei), during the manual update of the predictions to improve the model’s ability to discriminate tumour and non-tumour cells. The final nuclear model was trained using the combined set of labelled Ki-67 and PMS2 data, by the YOLOv5 architecture [40,41].

The cytoplasmic model was developed as previously described elsewhere [30], using 3060 tiles (40X, 800x800 pixels) from 34 WSIs from the PTEN-prostate set (Supplementary Table 7). Contours of more than 70000 tumour nuclei from the 3060 tiles were manually annotated and labelled as PTEN-positive or PTEN-negative. These were used as the ground truth in training and tuning of the model and were randomly split on the patient level into a training set (70 %) and a tuning set (30 %). The Mask R–CNN–network was trained to detect, delineate and classify tumour cells as either PTEN-positive or PTEN-negative. To improve the model, the detections that did not overlap with the manual annotations were manually reclassified into four classes: tumour PTEN-positive, tumour PTEN-negative, non-tumour PTEN-positive, or non-tumour PTEN-negative (102403 objects in total). The updated labelling of the dataset was used to train the final model.

The development set for the membranous model consisted of 292 tiles (40X, 1024x1024 pixels) from 25 WSIs from the β-catenin-prostate set. Contours of tumour cells from the 292 tiles were manually annotated and labelled as either β-catenin membranous positive or negative, resulting in 39254 labelled objects (Supplementary Table 7). These tiles were used as the ground truth in training and tuning of the model and were randomly split into a training set (75 %) and a tune set (25 %) at the patient level. The model was trained using the YOLOv5 architecture. For more details see Supplementary text.

#### 2.4.2 Internal test of deep learning models

Small test sets (tiles) were used to evaluate the DL models, and each set consisted of tiles from 25 randomly selected patients from each set (Graphical abstract, Supplementary Table 7). For the tiles from each set, positive and negative tumour cells were manually counted (point-annotated) by two observers using Manual Counter (In-house developed software, Supplementary Fig. 2). To avoid bias in the correlation analyses, four observers were involved in creating the development sets, and four observers (two not involved in the development set) provided manual counts for the test sets, designed as either observer 1 or 2 for each model (Supplementary Table 6). For the nuclear and membranous model, observer 1, who provided manual counts for the test sets, was partially involved in creating the development sets for these models. For the cytoplasmic model, observer 1 was solely responsible for making development set for the cytoplasmic model. None of the human experts designated as observer 2 were involved in creating development sets for either model. The counts generated by the DL models were compared to the manual counts in the test sets to identify the best performing models. The models with the highest correlation with manual counts in the test sets were applied to the full sets.

#### 2.4.3 Validation of deep learning models

The DL nuclear model was validated in the following data sets: Ki-67-prostate, Ki-67-breast, MSH6-colon and MSH6-endometrium, PMS2-endometrium, CCNB1-prostate, ER-breast and PR-breast. The cytoplasmic model was validated in the PTEN-prostate Cohort 2 and Mapre2-prostate sets, while the membranous model underwent validation in the CD44-prostate and Flotillin1-prostate sets. The cytoplasmic model was applied to automatically detected tumour areas in the PTEN-prostate and Mapre2-prostate datasets, as previously described [30]. For all other sets, the models were applied to manually annotated tumour areas.

The manual and DL scores were compared using correlation analyses in all sets. None of the human experts who provided manual scores for the validation sets for the nuclear and membranous models, designated as observer 1, participated in the development of these models (Supplementary Table 6). For the cytoplasmic model the manual scores in PTEN-prostate Cohort 2 and Mapre2-prostate sets were provided by the same human expert who was responsible for creating the development set. Additionally, survival analyses were performed to assess the relationship between protein expression, as determined by both manual and DL scores, and patient outcomes in the colon, endometrial, and prostate cancer datasets.

### 2.5 Statistical analyses

The R-squared coefficient was calculated in Microsoft Excel (Microsoft Corporation, Redmond, WA , USA) to measure the correlations between scores obtained by manual counting and DL models in the internal test sets. Bland-Altman plots were depicted using Python (v 3.8) (*statsmodels* v 0.14.0). Correlations between the dichotomized scores were evaluated using Pearson’s Chi-square test for categorical variables, κ-statistics (SPSS software v 26.0, IBM Corporation, NY, USA), and correct classification rate (CCR, the number of correct predictions/total predictions). For evaluation of the accuracy of DL models compared to manual counts considered as ground truth, the following measures were used: Precision = true positives/(true positives + false positives), Recall = true positives/(true positives + false negatives) and F1 score = 2 x recall x precision/(recall + precision). The ability of manual and DL scores to predict patient outcome was assessed using univariable survival analyses with cancer-specific survival (CSS) for the colon and endometrial sets, and time to recurrence (TTR) [42] for the prostate cancer set. Survival curves were depicted with the Kaplan-Meier method and differences between groups were compared using the Mantel-Cox Log Rank test (SPSS). Hazard ratios (HR) and 95 % confidence intervals (CI) were calculated by Cox regression analyses (SPSS) with dichotomized IHC scores as categorical variables. Two-sided p-values ≤0.05 were considered statistically significant.

## 3 Results

### 3.1 Performance of deep learning models in the training sets

The performance of the DL models in the tuning set from the training subset were evaluated, and the final nuclear DL model had a recall of 0.857 (train 0.934), precision of 0.693 (train 0.756), F1 score of 0.766 (train 0.836) and mean average precision of 0.798 (train 0.901) (see supplementary text for details). The cytoplasmic model had a recall of 0.688 (train 0.887), a precision of 0.750 (train 0.858), an F1 score of 0.718 (train 0.872), and a mean average precision of 0.716 (train 0.850). The membranous model had a recall of 0.663 (train 0.855), a precision of 0.676 (train 0.842), an F1 score of 0.669 (train 0.848), and a mean average precision of 0.517 (train 0.832).

### 3.2 Dichotomization of IHC scores and protein scores obtained by manual observers and DL

The proteins were categorized into two groups, as detailed in Table 1. For most markers, we adopted cut-off levels that had been previously described in the literature. For the remaining markers, quartiles or medians were selected, depending on the distribution of scores. The resulting cut-off values for manual and DL scores are specified in footnote in Table 1. The results obtained from both manual and DL scoring in the development set and validation sets are shown in Supplementary Tables 8 and 9, respectively.

**Table 1:** Cut-off levels used for dichotomizing protein markers.

Protein expression					
Protein	Tissue	Compartment	Low/Lost	High/Present	Reference
Ki-67	Colon	Nucleus	≤25 %	>25 %	[26]
Ki-67	Prostate	Nucleus	≤75th percentile	>75th percentile	[[25], [43]]
Ki-67	Breast	Nucleus	≤15 %	>15 %	[44]
PMS2	Colon	Nucleus	≤5 %	>5 %	[45]
PMS2	Endometrium	Nucleus	≤15 %	>15 %	Based on development set
MSH6	Colon	Nucleus	≤5 %	>5 %	[45]
MSH6	Endometrium	Nucleus	≤15 %	>15 %	Based on development set
CCNB1	Prostate	Nucleus	≤75th percentile*	>75th percentile	[28]
Oestrogen receptor	Breast	Nucleus	≤10 %	>10 %	[22]
Progesterone receptor	Breast	Nucleus	≤10 %	>10 %	[22]
PTEN	Prostate	Cytoplasmic	≤50 %	>50 %	[30]
Mapre2	Prostate	Cytoplasmic	<=median	>median	–
β-catenin	Prostate	Membranous	≤25th percentile	>25th percentile	–
CD44	Prostate	Membranous	≤25th percentile	>25th percentile	–
Flotillin1	Prostate	Membranous	<=median	>median	–

Abbreviations: CCNB1 - Cyclin B, MSH6 - MutS Homolog 6, PMS2 - PMS1 Homolog 2, Mismatch repair component, PTEN - Phosphatase and tensin homolog. *Thresholds: β-catenin-prostate; by the 25th percentile (95.0 % for manual scores, and 95.2 % for deep learning scores). Ki-67-prostate, by the 75th percentile (4 % for manual scores and 8.9 % for deep learning scores). CCNB1-prostate: by the 75th percentile (2 % for manual scores and 1.3 % for deep learning scores). Mapre2-prostate, by the median, (manual scores 99.5 % and for deep learning scores 95.5 %. CD44-prostate by the 25th percentile (Manual scores 5 % and 15.9 % for deep learning scores). Flotillin1-prostate by the median (manual scores 60 % and 84.3 % for deep learning scores).

### 3.3 Correlation between manual and DL counts

**Fig. 1:**
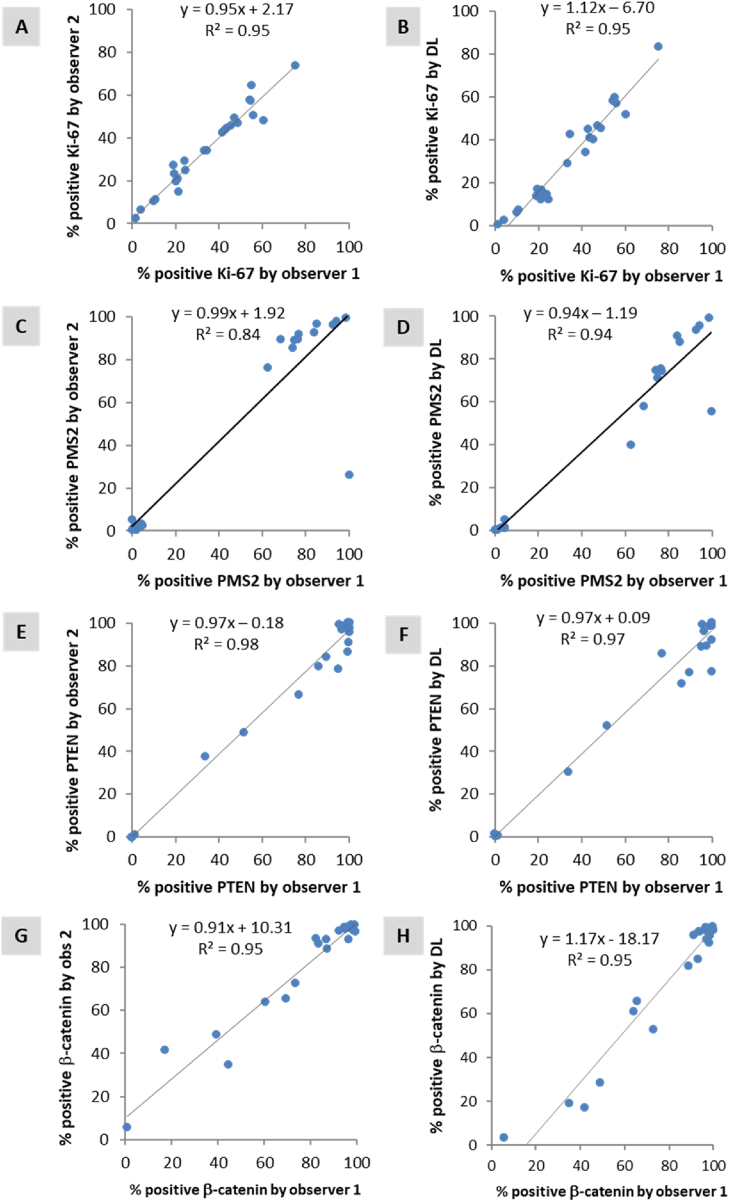
Scatter plots displaying the correlation between the scores assigned by observer 1 and observer 2 (A, C, E, G), as well as the correlation between observer 1 and the scores generated by deep learning models (B, D, F, H) in the test set for Ki-67-colon (A, B), PMS2-colon (C, D), PTEN-prostate (E, F) and β-catenin-prostate (G, H).

The results from the correlation analyses between manual counts provided by two observers (observer 1 and 2), and the scores generated by DL models for each internal test set are shown in Fig. 1. The correlations between observer 1 and 2 had an average correlation of R^2^ = 0.93 (range: 0.84–0.98). The correlations between observer 1 and DL had an average correlation of R^2^ = 0.95 (range: 0.93–0.96). Bland-Altman plots comparing manual counts by observer 1 and DL are shown in Supplementary Fig. 3.

**Fig. 2:**
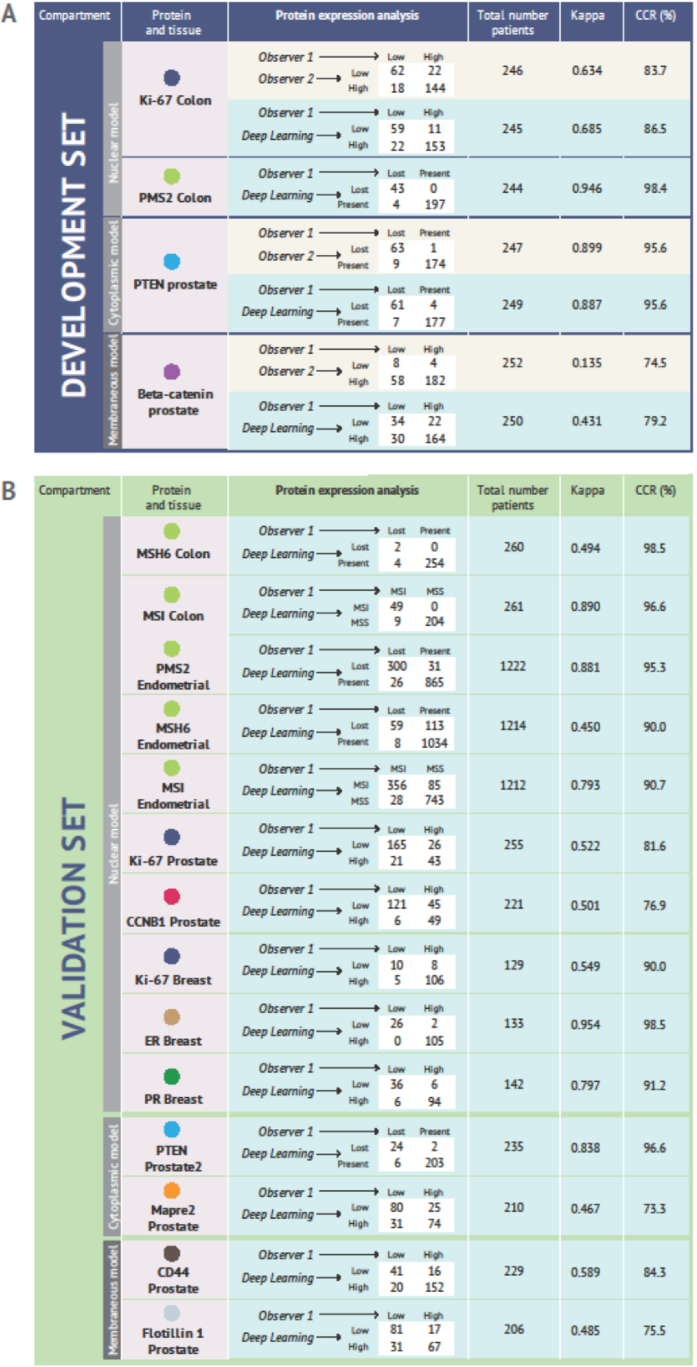
**A.** Agreement between the scores provided by manual observer 1 and observer 2, as well as scores assigned by observer 1 and those generated by deep learning models in the development sets. Assessment was performed by Pearson Chi-square test, Kappa statistics and the Correct Classification Rate (CCR). All values *p* < 0.001. **B.** Agreement between the scores assigned by observer 1 and those generated by deep learning models in the validation sets. Assessment was performed by Pearson Chi-square test, Kappa statistics and the Correct Classification Rate (CCR). All values *p* < 0.001.

The results from the correlation analyses comparing the dichotomized semi-quantitative scores provided by two observers and the scores generated by DL models in each development test subset and validation sets are shown in Fig. 2A and B, respectively. In the development sets the average CCR was 84.6 % (range: 74.3 % (β-catenin-prostate) to 98.5 % (PMS2-colon). For all materials in the development set, we observed a higher correlation between observer 1 and DL model predictions than between the two observers (Fig. 2A).

When comparing the results from manual scores and DL scores in the validation sets, we observed an average CCR of 91.5 % (range: 76.9–98.5 %) for the nuclear model, 85.6 % (range: 73.3–96.6 %) for the cytoplasmic model, and 78.4 % (range: 75.5–84.3 %) for the membranous model. The *p*-values were <0.001 for all correlations.

### 3.4 Evaluation of prognostic impact

#### 3.4.1 Nuclear model

**Fig. 3:**
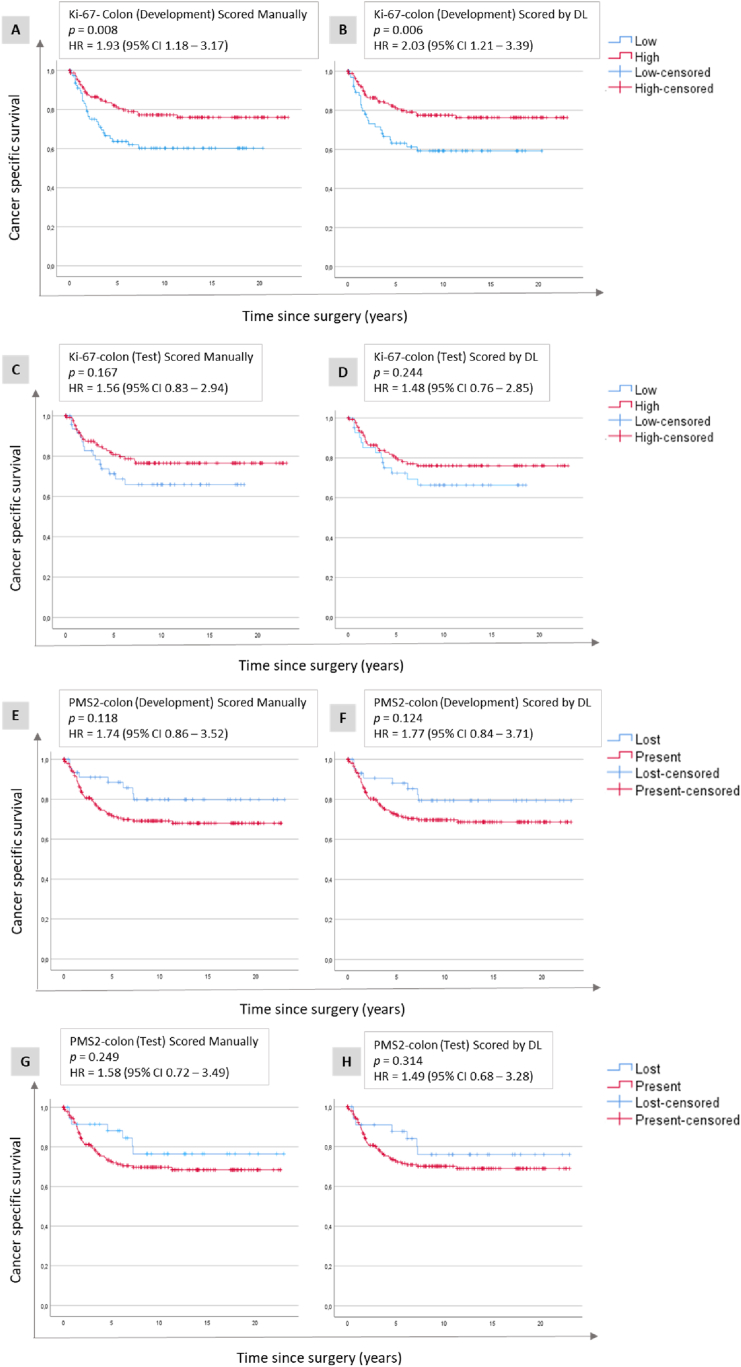
**Development and test of nuclear protein expression.** Kaplan-Meier plots illustrating cancer-specific survival related to Ki-67 (A, B, C, D) and PMS2 (E, F, G, H) expression in colon cancer. These plots are shown for both the development data set (A, B, E, F) and the internal test subset (C, D, G, H), with assessments performed using manual scores (A, C, E, G), and deep learning (DL) scores generated by the nuclear model (B, D, F, H). Abbreviations: CI = confidence interval; HR = hazard ratio.

In the Ki-67-colon set, used for development, patients with low Ki-67 expression had significantly shorter CSS compared to those with high Ki-67 expression when using Ki-67 scores obtained by either scoring method, and thresholds described in Table 1 (Fig. 3 (A and B), Table 2). Resulting in HR of 1.93 (95 % CI 1.18–3.17) for manual and 2.03 (95 % CI 1.21–3.39) for DL scores. In the internal test set, where the 69 patients (1 tile per patient) used in development were excluded, there was a similar trend but no significant separation of the groups by either manual or DL scores (Fig. 3 (C and D), Table 2). Also, in the PMS2-colon set, used for identification of positive controls in the development set, we observed similar separation between groups for both manual and DL scoring methods, but the differences were not statistically significant (Fig. 3 (E, F, G and H), Table 2).

**Table 2:** Summary of univariable survival analyses for all analyzed sets and methods.

		Protein and material	Method	Low, lost MSI (n)	High, present or MSS (n)	*p-*value	Hazard ratio	95 % confidence interval HR
Development test dataset	Nuclear	Ki-67-Colon (high vs low) (n = 251)	Observer 1	84	167	0.008	1.93	1.18–3.17
			Observer 2	84	162	0.021	1.80	1.08–3.00
			Deep learning	70	175	0.006	2.03	1.21–3.39
Internal test subset		Ki67-Colon (high vs low) (n = 180)	Observer 1	52	128	0.167	1.56	0.83–2.94
			Deep learning	44	136	0.244	1.48	0.76–2.90
Development test dataset		PMS2-colon (lost vs present) (n = 260)	Observers 1 and 2	53	206	0.118	1.74	0.86–3.52
			Deep learning	47	215	0.124	1.77	0.84–3.71
Internal test subset		PMS2-colon (lost vs present) (n = 221)	Observers 1 and 2	36	185	0.249	1.58	0.72–3.49
			Deep learning	35	201	0.314	1.49	0.68–3.28
Internal test subset	Cytoplasmic	PTEN-prostate (present vs lost) (n = 253)	Observer 1 and 2	68	181	<0.001	2.41	1.57–3.70
			Deep learning	65	184	0.002	1.96	1.27–3.02
Development test dataset	Membranous	β-catenin (high vs low) (n = 252)	Observer 1 and 2	69	183	0.092	1.49	0.93–2.39
			Deep learning	63	189	<0.001	2.13	1.35–3.37
Internal test subset		β-catenin (high vs low) (n = 227)	Observer 1 and 2	58	169	0.053	1.66	0.99–2.78
			Deep learning	54	173	<0.001	2.37	1.44–3.91
Validation data sets	Nuclear	MSH6-colon (lost vs present) (n = 262)	Observers 1 and 2	6	254	0.150	21.11	0.04-∞
			Deep learning	2	260	0.404	NA	NA
		MSI-Colon (MSI vs MSS) (n = 262)	Observers 1 and 2	58	204	0.039	2.06	1.02–4.17
			Deep learning	49	213	0.086	1.89	0.90–3.96
		PMS2-endometrium (lost vs present) (n = 1224)	Observer 1	326	895	0.016	1.44	1.07–1.95
			Deep learning	334	890	0.017	1.43	1.07–1.92
		MSH6-endometrium (lost vs present) (n = 1224)	Observer 1	67	1146	0.036	2.09	1.03–4.28
			Deep learning	183	1041	<0.001	2.65	1.64–4.28
		MSI-endometrium (MSI vs MSS) n = 1223)	Observer 1	384	827	<0.001	1.66	1.24–2.21
			Deep learning	453	770	<0.001	1.89	1.43–2.49
		Ki-67-prostate (low vs high) (n = 256)	Observer 1	186	70	0.005	1.92	1.21–3.05
			Deep learning	191	64	0.012	1.81	1.13–2.90
		CCNB1-prostate (low vs high) (n = 221)	Observer 1	127	94	<0.001	2.30	1.45–3.65
			Deep Learning	166	55	0.002	2.10	1.30–3.38
	Cytoplasmic	PTEN-prostate2 (present vs lost) (n = 255)	Observer 1	50	205	<0.001	3.34	2.06–5.39
			Deep Learning	47	212	<0.001	3.32	2.04–5.38
		Mapre2-prostate (high vs low) (n = 225)	Observer 1	115	114	0.014	1.74	1.11–2.72
			Deep learning	108	108	0.054	1.55	0.99–2.41
	Membranous	CD44-prostate (high vs low) (n = 229)	Observer 1	60	169	0.056	1.56	0.99–2.47
			Deep Learning	56	166	0.007	1.89	1.18–3.02
		Flotillin1-prostate (high vs low) (n = 210)	Observer 1	120	89	0.005	2.12	1.23–3.63
			Deep Learning	105	105	0.023	1.75	1.07–2.85

Abbreviations: CCNB1 - Cyclin B, HR - hazard ratio, MSH6 - MutS Homolog 6, MSI - microsatellite instable, MSS - microsatellite stable, PMS2 - PMS1 Homolog 2, Mismatch repair component, NA - not available, PTEN - Phosphatase and tensin homolog, WSI - whole slide images.

The nuclear model was validated in the MSH6-colon set, and the correlation between manual and DL scoring was good (98.5 %, Fig. 2B), but Cox regression analyses did not converge, likely because of very few cases with lost MSH6 (n = 6 manual and n = 2 for DL) (Fig. 4a (A and B), Table 2). When combining PMS2 and MSH6 scores into MSI assessment, we observed that patients with MSS tumours had significantly shorter CSS compared to those with MSI tumours when using manual scores (HR = 2.06, 95 % CI 1.02–4.17), (Fig. 4a (C)–Table 2). A similar trend was observed for DL scores (Fig. 4a (D)), but the difference in CSS between the MSS and MSI groups did not reach statistical significance (HR = 1.89, 95 % CI 0.90–3.96), (Table 2). Validation in the Ki-67-prostate set showed that patients with high Ki-67 had significantly shorter TTR compared to patients with low Ki-67 tumours for both manual (HR = 1.92, 95 % CI 1.21–3.05) and DL scores (HR = 1.81, 95 % CI 1.13–2.90), (Fig. 4a (E and F), Table 2). In the CCNB1-prostate set, patients with high CCNB1 had significantly shorter TTR compared to those with high CCNB1 tumours for both manual scores (HR = 2.30, 95 % CI 1.45–3.65), and DL scores (HR = 2.10, 95 % CI 1.30–3.38), (Fig. 4a (G and H), Table 2). The nuclear model was further validated in MSH6 and PMS2 in endometrial cancer, where loss of expression was significantly associated with longer CSS for both manual and DL scores. For the presence of MSH6, the respective HRs were 2.09 (95 % CI 1.03–4.28) and 2.65 (95 % CI 1.64–4.28) (Fig. 4b (A and B), while for PMS2, the HRs were 1.44 (95 % CI 1.07–1.95) and 1.43 (95 % CI 1.07–1.92), (Fig. 4b (C and D)). For a combination of MSH6 and PMS2 into MSI status, the HRs for MSS were 1.66 (95 % CI 1.24–2.21) for manual scores, and 1.89 (95 % CI 1.43–2.49) for DL scores (Fig. 4b (E and F), Table 2).

**Fig. 4a:**
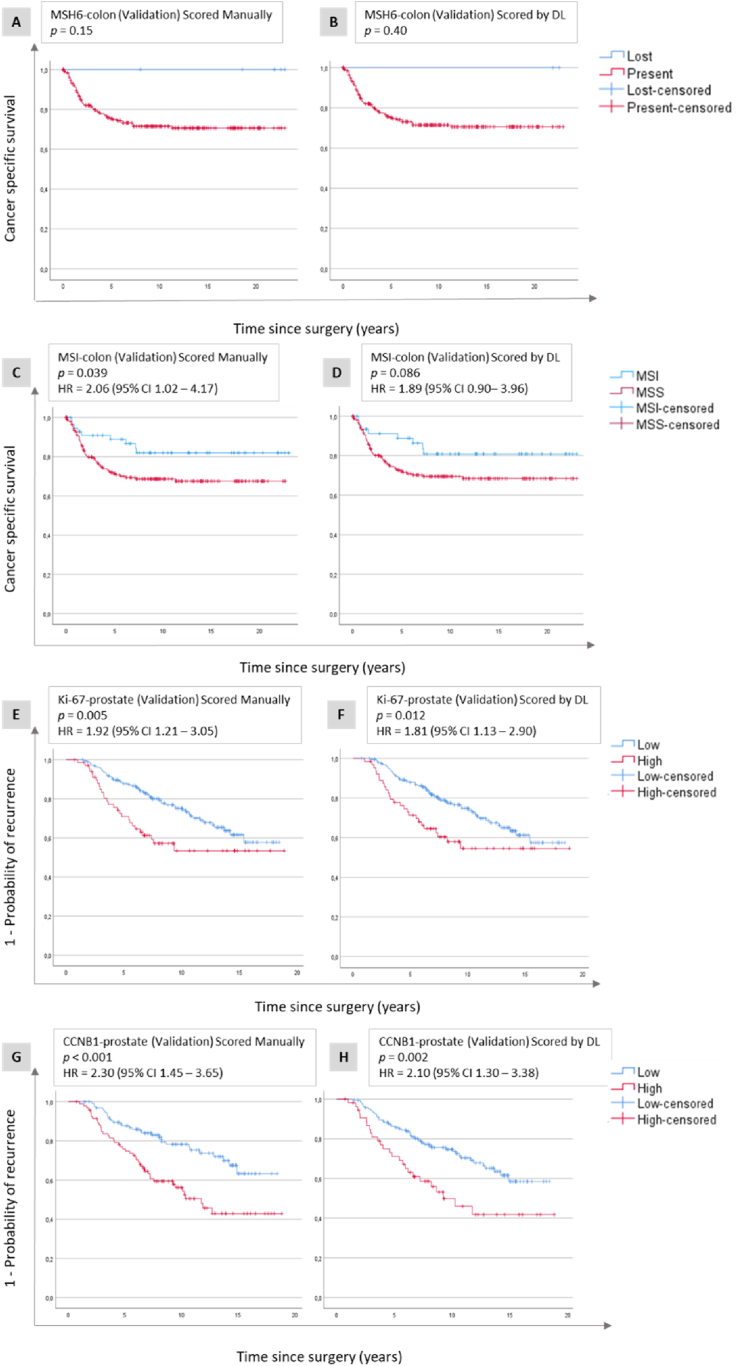
**Validation of nuclear protein expression.** Kaplan-Meier plots illustrating cancer-specific survival related to MSH6 expression (A and B) and MSI status (C and D) in colon cancer as well as Ki-67 (E and F) and CCNB1 (G and H) expression in prostate cancer. These plots are shown for the validation data sets, with assessments performed using manual scores (A, C, E, G), and deep learning (DL) scores generated by the nuclear model (B, D, F, H). Abbreviations: CI - confidence interval, HR - hazard ratio, MSI - microsatellite unstable, MSS - microsatellite stable.

**Fig. 4b:**
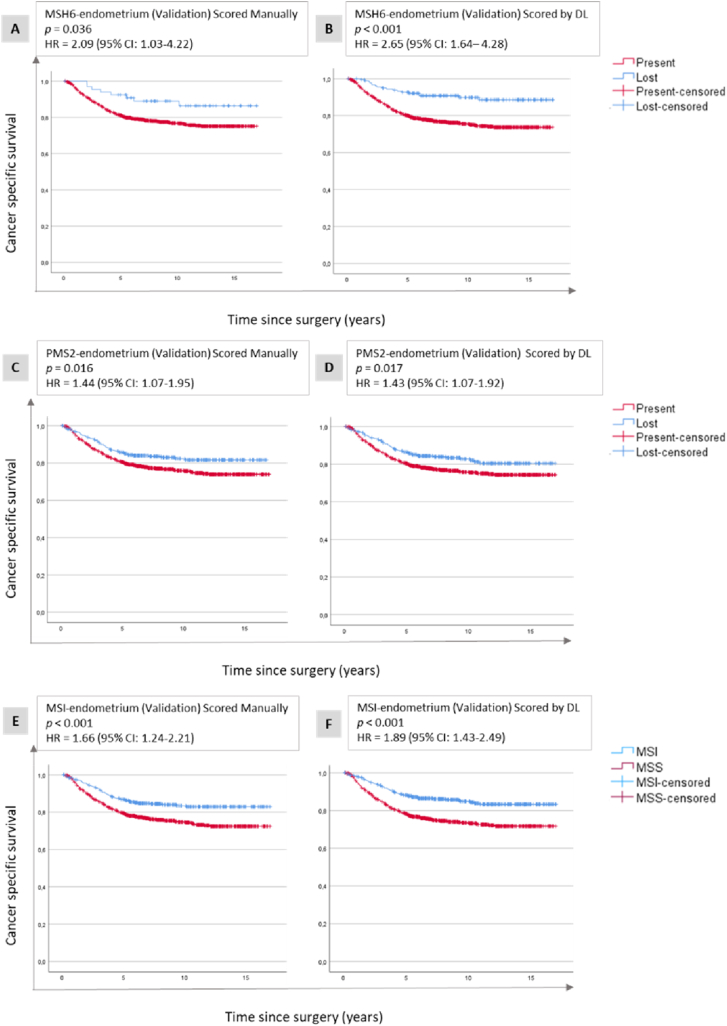
**Validation of nuclear protein expression.** Kaplan-Meier plots illustrating cancer-specific survival related to MSH6 (A and B) and PMS2 (C and D) expression as well as MSI status (E and F) in endometrial cancer. These plots are shown for the validation data sets, with assessments performed using manual scores (A, C, E), and deep learning (DL) scores generated by the nuclear model (B, D, F). Abbreviations: CI - confidence interval, HR - hazard ratio, MSI - microsatellite unstable, MSS - microsatellite stable.

#### 3.4.2 Cytoplasmic model

**Fig. 5:**
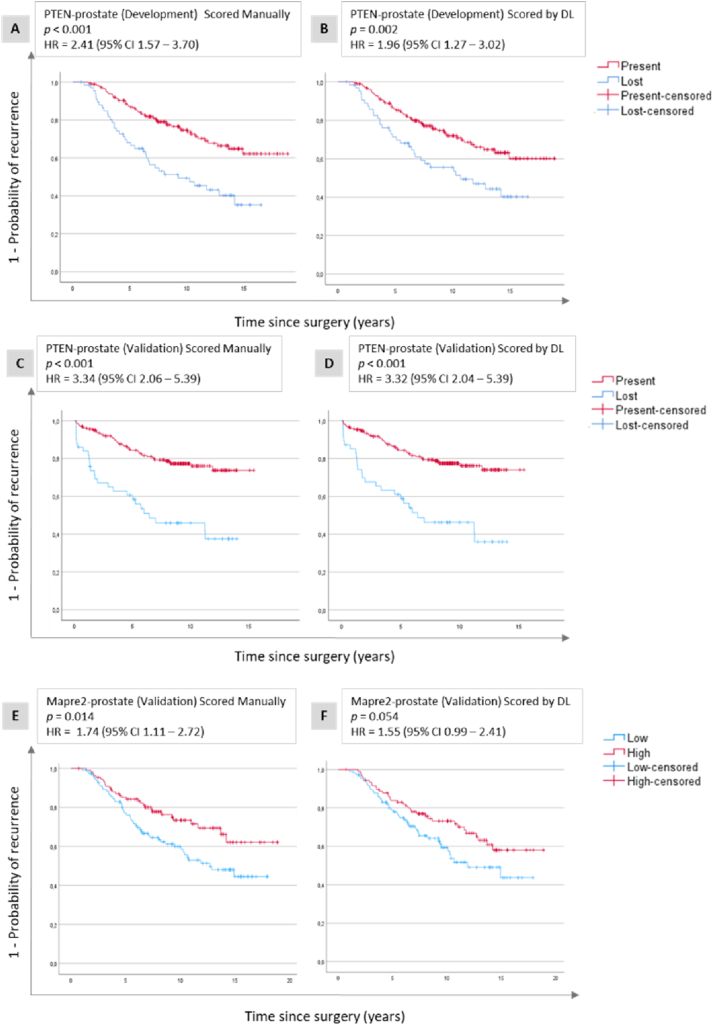
**Development and validation of cytoplasmic protein expression.** Kaplan-Meier plots illustrating time to recurrence related to PTEN (A, B, C, D) and Mapre2 (E and F) expression in prostate cancer. These plots are shown for both the development data set (A and B) and the validation data sets (C, D, E, F), with assessments performed using manual scores (A, C, E), and deep learning (DL) scores generated by the cytoplasmic model (B, D, F). Abbreviations: CI = confidence interval; HR = hazard ratio.

In the PTEN-prostate set used for development, patients with PTEN-loss had significantly shorter TTR compared to those with PTEN-present tumours for both manual scores (HR = 2.41, 95 % CI 1.57–3.70) and DL scores (HR = 1.96, 95 % CI 1.27–3.02) (Fig. 5 (A and B), Table 2). Validation in the independent prostate Cohort 2, showed similar results (manual scores: HR = 3.34, 95 % CI 2.06–5.39 and DL scores: HR = 3.32, 95 % CI 2.04–5.38) (Fig. 5 (C and D), Table 2). The model was additionally validated in Mapre2-prostate, and for both manual and DL scores patients with low expression of Mapre2 had shorter TTR than patients with high Mapre2 with HR = 1.74 (95 % CI 1.11–2.72) for manual scores, and HR = 1.55 (95 % CI 0.99–2.47) for DL scores (Fig. 5 (E and F), Table 2).

#### 3.4.3 Membranous model

**Fig. 6:**
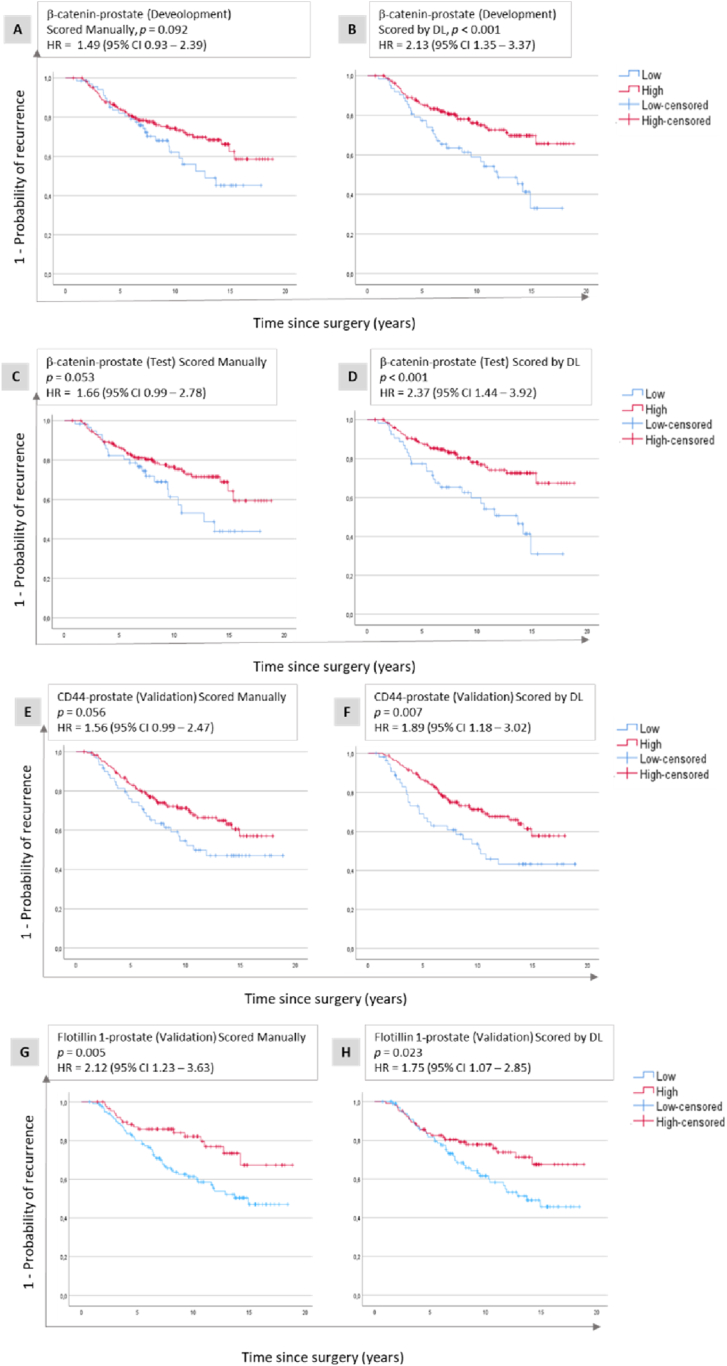
**Development, test and validation of membranous protein expression.** Kaplan-Meier plots illustrating time to recurrence related to membranous β-catenin (A, B, C, D), CD44 (E and F) and Flotillin1 (G and H) expression in prostate cancer. These plots are shown for the development data set (A and B), the internal test subset (C and D) and the validation data sets (E, F, G, H), with assessments performed using manual scores (A, C, E, G), and deep learning (DL) scores generated by the membranous model (B, D, F, H). Abbreviations: CI = confidence interval; HR = hazard ratio.

In the β-catenin-prostate set used for development, patients with tumours with low β-catenin expression did not have significantly shorter TTR compared to patients with high β-catenin expression tumours when scored manually (HR = 1.49, 95 % CI 0.93–2.39, Fig. 6 (A), Table 2), but this association was significant when using DL scores (HR = 2.13, 95 % CI 1.35–3.37, Fig. 6 (B)–Table 2). Similar results were observed in the internal test subset (Fig. 6 (C and D), Table 2).

The membranous model was validated in CD44-prostate, where patients with tumours with low CD44 expression was associated with shorter TTR than patients with high CD44. Manual scores demonstrated an association with TTR close to the threshold of significance (HR = 1.56, 95 % CI 0.99–2.47, Fig. 6 (E)–Table 2), whereas DL scores provided a significant association (HR = 1.89, 95 % CI 1.18–3.02, Fig. 6 (F)–Table 2). When validated in Flotillin1-prostate, both manual (HR = 2.12, 95 % CI 1.23–3.63) and DL scores (HR = 1.75, 95 % CI 1.07–2.85) showed a significant shorter TTR for patients with tumours with low Flotillin1 (Fig. 6 (G and H), Table 2).

### 3.5 Correlation and survival analyses using additional thresholds for determining MSI status

In the reported DL results, a positive fraction of 5 % was used as threshold to assess the presence of PMS2 and MSH6 in colon cancer. In addition, we investigated thresholds of 10 %, 15 %, and 20 %. Thresholds of 15 % and 20 % showed a better correlation between manual scores and DL scores (CCR at 15 % was 98.5 % for DL and CCR at 20 % was 98.9 % for DL) (Supplementary Table 10), compared with using the initial 5 % threshold (CCR 96.6 % for DL). Using 15 % and 20 % as thresholds also resulted in better discrimination between patients with MSI and MSS tumours in survival analyses for DL scores (HR = 1.99, 95 % CI 0.99–4.03, *p* = 0.049 and HR = 1.92, 95 % CI 0.98–3.77, *p* = 0.053, respectively), (Supplementary Fig. 4, Supplementary Table 11).

## 4 Discussion

We developed and validated cell-level DL models for the analysis of WSIs of tissue sections from four cancer types that were IHC-stained for 11 markers which spanned common staining patterns: nuclear, cytoplasmic, and membranous. We have shown that scores generated by DL models were highly correlated with manual scores and provided similar results in survival analyses in terms of HRs and *p*-values. Furthermore, we observed that the DL models developed for a specific marker within one cancer type could be applied to other datasets encompassing diverse protein markers, tissue types, and laboratory settings. This suggests that these models have the capacity to generalize across various data sources and perform effectively on previously unseen data - an essential criterion for the clinical integration of such techniques [15].

Certain characteristics are unique to specific proteins and tissue types, necessitating adjustments to the initial models for accurate detection. To develop the nuclear model using the Ki-67-colon set, we labelled only positive and negative tumour cells in the development set. The model showed a good correlation with manual counts in the tuning and test sets, as well as semi-quantitative scores in the development set for Ki-67-colon. But when this model was applied to the PMS2-colon test set, we observed that internal positive controls (i.e. non-tumour cells) were wrongly classified as true positives, thus confounding the PMS2 scores. This was not an issue for the Ki-67-colon set, where few non-tumour cells were positive. Furthermore, given the relatively high Ki-67 expression, the presence of false positives had minimal impact on the total score. The inclusion of internal positive controls as a third class in PMS2 labelling, in addition to positive and negative tumour cells, improved the model’s ability to distinguish between tumour and non-tumour cells and resulted in a stronger correlation between the DL score and manual cell counts in the PMS2-colon test set, and likely improved generalizability of the model.

One of the strengths of this study is the inclusion of five retrospective patient cohorts with a relatively large number of patients, where the different scoring methods were evaluated both by correlation with manual scores and by prognostic impact. Furthermore, we used separate internal test sets to compare predictions from each model with manual counts. This allowed us to do additional training to improve the performance of the models before applying them to the full sets. To avoid bias in the correlation and survival analyses and to ensure an unbiased model, a panel of nine observers participated in creating the development and tuning sets, as well as manual counting in test sets and manual scoring for validation set. The cytoplasmic model was the only one where the same observer was responsible for creating the development set and providing manual scores in the test and validation sets. However, the manual PTEN scores of this observer and those of observer 2 in the development set exhibited a strong correlation, and the CCRs between the two observers, as well as for observer 1 and the DL scores, were identical. For the nuclear and membranous models, we observed a stronger correlation between observer 1 and the DL scores compared to the correlation between the two observers in the development set. This discrepancy could be due to the fact that the human expert designated as observer 1 was partially involved in creating the development sets and provided manual counts for the test sets for these models. However, it’s important to emphasize that for both models, manual scores in the development test subsets and validation sets were provided by different human experts than those who were involved in the development of these models. In addition, our study was performed using WSIs from routine tissue sections representing the entire specimen in the tissue block, whereas many previous studies were performed using tissue microarray (TMA) slides [46,47]. Although TMAs are convenient in the research setting as they assemble many small tumour tissue samples from different patients on a single histologic slide, they may not accurately represent clinical practice. Another strength of our study is using supervised learning and cell-based algorithms, rather than weakly supervised learning with imprecise labels that is not as easily explainable.

For the colon, prostate and endometrial cohorts, we had long-term follow-up data, enabling evaluation of protein scoring by prognostic impact in survival analysis. Previous studies evaluating digital image analysis methods with survival analyses are scarce. In agreement with findings from others [11], we show excellent reproducibility for the detection of Ki67, with reliable scores predicting outcomes for patients with colon and prostate cancer. A recent study by Fan et al. [48], in agreement with our study show high sensitivity for using deep learning for scoring of Ki-67, ER and PR in breast carcinoma. However, many studies relied solely on agreement with manual scoring [5,46,[49], [50], [51]], which may be insufficient due to inter- and intra-observer variability in manual scoring [2].

A lower level of agreement with the manual scores does not always translate into poorer model performance when it comes to associating the results with patient outcomes. We observed that among the analyzed sets, the β-catenin-prostate set had one of the poorest agreements comparing scores obtained by the two observers (CCR = 74.5 %) as well as observer 1 and DL model (CCR = 79.2 %). However, the DL scores provided statistically significant stratification of patients in survival analyses (*p* < 0.001), while manual scores did not (*p* = 0.092). This discrepancy could be attributed to the challenge of detecting membranous staining with the human eye, suggesting that the membranous model could potentially offer greater accuracy. In addition, we have observed that our DL models perform less effectively when applied to prostate cancer samples compared to those of colon, breast and endometrial cancer samples. It is reasonable to consider that including prostate cancer samples in the training set during model development would improve their performance.

We used predefined thresholds for dichotomizing scores from both scoring methods for each set to avoid the problem of multiple testing. For most proteins and cancer types we adopted cut-off values already established in the literature. However, for proteins like Mapre2 and Flotillin1, on which fewer studies were published, we opted to determine cut-off values based on either the median or quartiles, depending on the distribution of scores. It is important to note that these cut-off values may not necessarily be applicable to other datasets examining the prognostic value of these proteins. To identify the optimal cut-off points that offer clinically meaningful risk stratification of patients, further studies with independent validation cohorts are necessary.

The manual scores, assessed through semi-quantitative estimation, are prone to uncertainty and may not align directly with the actual counts generated by DL models. PMS2 and MSH6 were scored manually as “lost” or “present”, without estimating the percentage of negative tumour cells. Initially, PMS2 and MSH6 scores generated by the DL model were categorized using the 5 % threshold, based on existing literature [45]. While manual scores provided significant prognostic information (MSS had shorter CSS than MSI) (*p* = 0.039, 95 % CI 1.02–4.17), DL scores showed borderline significance (*p* = 0.086, 95 % CI 0.90–3.96). We hypothesized that by using a higher threshold, we could account for positive internal controls (non-tumour cells expressing PMS2 or MSH6) that were erroneously classified as true positives. Therefore, additional correlation and survival analyses using 10 %, 15 %, and 20 % thresholds were performed in the development test set. Increasing the threshold to 15 %, we observed a statistically significant stratification of the two patient groups using DL scores (*p* = 0.049). Based on these observations, we set the thresholds for categorizing PMS2 and MSH6 in the endometrium to 15 %.

Our study has some limitations. First, our study is retrospective, necessitating validation of the models in real-world data before implementation. In addition, both the cytoplasmic and membranous DL models are developed using only one protein and cancer type. We consider that incorporating multiple protein markers and cancer types into the development set could enhance the models’ performance and generalizability. Furthermore, we only implemented automatic tumour detection for the cytoplasmic model [30]. Integration of automatic tumour detection could facilitate fully automated scoring, expediting the utilization of DL models. Lastly, we only compared the DL scores with manual scores, without evaluating them against scoring platforms.

The adoption of automated IHC scoring methods offers a valuable solution to address the current shortage of pathologists and the growing diagnostic workload [52]. These methods have the potential to streamline the diagnostic process, ultimately leading to more timely diagnoses for patients. Furthermore, automated approaches ensure reproducibility, providing consistent results. However, it should be acknowledged that the accuracy of DL models depends on the availability of a substantial volume of well-labelled and meticulously annotated training data. The process of training DL models is time-intensive, involving the evaluation and training of multiple configurations to identify the most suitable model for a given problem. This requires proficiency in deep learning techniques and access to powerful computers equipped with graphical processing units. However, since the DL models are generalizable we consider the effort acceptable.

## 5 Conclusion

The inevitable integration of digital image analysis is set to enhance pathology workflows, effectively addressing the growing number of diagnostic cases and the shortage of pathologists. Our study findings strongly emphasize the feasibility of automated IHC scoring with DL models, serving as an adequate alternative for the conventional manual scoring performed by pathologists. Although the development of DL models requires substantial resources, their capability to generalize across diverse data sets may expedite their clinical integration.

## Funding

This research did not receive any specific grant from funding agencies in the public, commercial, or not-for-profit sectors.

## Data availability

All labelled training data and code will be made available on request.

## CRediT authorship contribution statement

**Wanja Kildal:** Conceptualization, Data curation, Formal analysis, Investigation, Methodology, Project administration, Visualization, Writing – original draft, Writing – review & editing. **Karolina Cyll:** Formal analysis, Investigation, Writing – original draft, Writing – review & editing. **Joakim Kalsnes:** Formal analysis, Investigation, Methodology, Software, Validation, Writing – review & editing. **Rakibul Islam:** Investigation, Visualization, Writing – review & editing. **Frida M. Julbø:** Investigation, Methodology, Software, Validation, Writing – review & editing. **Manohar Pradhan:** Investigation, Writing – review & editing. **Elin Ersvær:** Conceptualization, Investigation, Methodology, Writing – review & editing. **Neil Shepherd:** Resources, Writing – review & editing. **Ljiljana Vlakovic:** Investigation, Writing – review & editing. **OSBREAC:** Resources, reviewed and edited the manuscript. **Xavier Tekpli:** Writing – review & editing. **Øystein Garred:** Resources, Writing – review & editing. **Gunnar B. Kristensen:** Resources, Writing – review & editing. **Hanne A. Askautrud:** Resources, Writing – review & editing. **Tarjei S. Hveem:** Conceptualization, Formal analysis, Methodology, Software, Supervision, Validation, Writing – review & editing. **Håvard E. Danielsen:** Conceptualization, Funding acquisition, Resources, Supervision, Writing – review & editing.

## Declaration of competing interest

The authors declare that they have no known competing financial interests or personal relationships that could have appeared to influence the work reported in this paper.
