# Supplementary material for: Deep learning for automated scoring of immunohistochemically stained tumour tissue sections – Validation across tumour types based on patient outcomes
Source: Heliyon. 2024 Jun 13;10(13):e32529. doi: 10.1016/j.heliyon.2024.e32529 (PMC11261074; doi:10.1016/j.heliyon.2024.e32529)
Supplement: Multimedia component 1 [file mmc1.docx]

**Supplementary material**

**Supplementary text**

- 1. **Figure legends**

**Graphical abstract**

Footnote: Abbreviations: MSI – microsatellite instable, PMS2 - PMS1 Homolog 2, Mismatch repair component, MSH6 - MutS Homolog 6, PTEN - Phosphatase and tensin homolog. CCNB1 – Cyclin B, ER – Estrogen receptor, PR – Progesterone receptor, WSI – whole slide images. For MSI evaluation both PMS2 and MSH6 were stained for.

**Supplementary Figure 1.** Consort diagrams showing each patient cohort and protein.

Footnote: Abbreviations: IHC - immunohistochemistry, OUH – Oslo University Hospital, Osl2 - Oslo 2 study, MSI - microsatellite instable, PMS2 - PMS1 Homolog 2, Mismatch repair component, MSH6 - MutS Homolog 6, n= number of patients, PTEN - Phosphatase and tensin homolog. CCNB1 - Cyclin B, ER - Estrogen receptor, PR - Progesterone receptor. **A consecutive series. ***Three blocks per patient were analyzed.

**Supplementary Figure 2.** Representative examples of expression patterns for all the included proteins and materials. The left panel shows the original image. In the middle panel, positive (green) and negative (red) objects were manually identified by human experts, while in the right panel, they were identified by deep learning models.

2.1. From top to bottom: Ki-67-colon, PMS2- colon, MSH6-colon, Ki-67-prostate, CCNB1-prostate.

2.2. From top to bottom: Ki-67-breast, ER-breast, PR-breast, MSH6-endometrium, PMS2-endometrium.

2.3. From top to bottom: PTEN-prostate, Mapre2-prostate, β-catenin-prostate, CD44-prostate, Flotillin1-prostate.

*Footnote: Abbreviations: Ki-67 - Marker of proliferation Ki-67, PMS2 - PMS1 Homolog 2, Mismatch repair component, MSH6 - MutS Homolog 6, PTEN - Phosphatase and tensin homolog, CCNB1 – Cyclin B, ER – Estrogen receptor, PR- progesterone receptor.*

**Supplementary Figure 3.** Bland Altman plot illustrating agreement between manual and deep learning counts in selected tiles from 24 patients in the test set for the proteins Ki-67, PMS2, PTEN and β-catenin.

Footnote: Abbreviations: DL – deep learning, SD – standard deviation, mean diff – mean difference.

**Supplementary Figure 4.** Kaplan-Meier plots illustrating cancer-specific survival related to the MSI status (combined PMS2 and MSH6) in colon cancer in the validation cohort, as determined by deep learning (DL) scores. The MSI status was classified as lost or present by using ≤5% (A), ≤10% (B), ≤15% (C) and ≤20% (D) thresholds. Abbreviations: MSI= microsatellite instability. CI = confidence interval; HR = hazard ratio.

**Protein expression and manual scoring**

Figure 1 present representative examples of IHC expression for proteins included in the study. Ki-67 was expressed in the nuclei of proliferating tumour and non-tumour cells, and IHC staining intensity varied from weak to strong). CCNB1 was expressed in the nuclei and cytoplasm of proliferating tumour and non-tumour cells, and IHC staining varied from moderate to strong. PTEN was expressed in the cytoplasm and nuclei of tumour and non-tumour cells. PTEN IHC staining was diffuse with varied intensity, mostly weak to moderate, and staining of similar intensity was often present in the stroma. Cells were considered PTEN-negative if the cytoplasmic and nuclear staining was absent or decreased compared to benign epithelial and/or stromal cells, which were used as positive controls (Cyll et al., 2021). β-catenin was expressed in the membrane of tumour and non-tumour cells, and, in addition, in the nuclei and cytoplasm in a subset of cells. β-catenin IHC staining varied from moderate to strong. Cells were considered β-catenin-negative if the membranous staining was covering <60% of the cell’s membrane; benign epithelial cells were used as positive controls. PMS2 and MSH6 were expressed in the nuclei of tumour and non-tumour cells. Their IHC-staining was either homogeneously positive or negative in nearly all WSIs and was scored as present or lost, without estimating the percentage of positive tumour cells. The adjacent immune cells served as internal controls. CD44 was expressed in the cytoplasm and membrane of tumour cells and benign epithelium, and also in immune cells and nerve fibres in the stroma. Flotillin1 was expressed in the cytoplasm and membrane of tumour cells and benign epithelium. Mapre2 was expressed in the cytoplasm and nucleus of tumour cells and benign epithelium, and immune cells and nerve fibres in the stoma were strongly positive. In some areas, Mapre2 was completely lost in the tumour glands, whereas the surrounding controls were positive. Both ER and PR were mainly expressed in the nuclei, but in some cases also in the cytoplasm, of breast cancer cells.

**Description of deep learning method**

**Development**

Training for both the nuclear and membranous models was performed on a standard computer with Linux operating system and 1 Nvidia Titan X (Pascal) GPU card with 12 GB memory. We used YOLOv5l model (the deep learning system used in this study is based on the open source YOLOv5 (<https://github>.com/ultralytics/yolov5/issues/1333) implementation by Ultralytics (Redmon et al., 2016; Redmon & Farhadi, 2018). YOLO (You Only Look Once) is a neural network architecture designed for object detection, *i.e.,* the computer vision task of locating and classifying objects in an image. The network takes an image as input and outputs object detections as bounding boxes with corresponding class labels and scores representing the certainty of the model. The model was initialized with weights pre-trained on the COCO dataset (<https://arxiv.org/abs/1405.0312>), downloaded from the Ultralytics yolov5 github repository. We used an image size of 800 or 1024 and a batch size of 8, and we trained for 100 epochs. Default hyperparameters were used. The reasons for going from an image size of 800 to 1024, was based on that manual evaluation of tumor content was easier on 1024 images.

A number of data augmentation processes were included in the default training setting: Mosaic Augmentation (4 images mixed into one single image), flip left-right (probability 0.5), hue, saturation and value augmentation in hsv space, scaling and translation. Colour augmentations were done on the mosaic image, rather than on each individual image.

The cytoplasmic model was trained on a machine with 4 Nvidia Titan Xp GPU cards. It consists of a tumour detector model and an object classifier model. The training and tuning of both models are described in detail in the supplementary of [https://www.mdpi.com/2072-6694/13/17/4291/htm]. Breifly, the tumour detector is a model based on Inception v3 [https://www.computer.org/csdl/proceedings-article/cvpr/2016/8851c818/12OmNvrMUfG], a classification convolution neural network. The input to the network is a tile with size 800x800 pixels, and the output is a classification as either tumour or non-tumour. The object classifier model was developed using the Mask R-CNN instance segmentation network [https://ieeexplore.ieee.org/document/8372616]. Input to the network is a tile with size 800x800 pixels. First, the input image is processed by a backbone convolutional neural network that extracts feature maps of the input image. Processing continues through two additional stages. The first stage generates object proposals in the shape of bounding boxes, i.e. rectangular candidate regions potentially containing a target object. The second stage predicts the class labels of the proposed objects, refines the bounding boxes and generates segmentation masks on pixel level for the proposed objects.

A detection was considered correct when the intersection over union (IoU) was above 0.5. The IoU was found by dividing the area of overlap between the ground truth bounding box and the predicted bounding box with the area of union:

IoU = $\frac{area of overlap}{area of union}$

We only allowed one detection per ground truth object to be classified as correct. If there were more than one detection per ground truth object, the other detections were classified as false even though IoU was above 0.5. Based on the IoU, we calculated the precision, recall, F1 score and mean average precision to evaluate our model.

Precision is the ratio of correct predictions (true positives) to total predictions:

Precision = $\frac{true positives}{true positives + false positives}$

Recall is the ratio of correct predictions to all ground truth objects:

Recall = $\frac{true positives}{true positives + false negatives}$

F1 score combines both recall and precision, and measures the weighted average of these two:

F1 score = $\frac{2 * recall * precision}{recall + precision}$

The average precision (AP) is found by calculating the area under the precision-recall curve, and the mean average precision is the average of AP across all the classes.

**Inference**

The process of analysing a new WSI is as follows:

1. The WSI at full resolution (40X) is split into adjacent, non-overlapping tiles by defining a grid of candidate tiles, starting at the upper left corner of the WSI. Tile size is 1024x1024 pixels for the nuclear and membranous model, and 800x800 pixels for the cytoplasmic model. The candidate tiles are read into Python as an RGB images using Openslide. Background tiles are excluded from further considerations. A tile is considered a background tile if more than 50% of the pixels in that tile has a grayscale value higher than 220. The remaining candidate tiles, i.e. not background tiles, are classified as either tumour or non-tumour based on:
   1. Manual tumour annotations. If the center position of the tile is inside the tumour annotation, the tile is classified as a tumour tile. Otherwise, it is classified as a non-tumour tile. This method is used for the nuclear and membranous models.
   2. The tumour detector network. Each candidate tile is passed through the tumour detector network and given a label as either tumour or non-tumour. This method is used for the cytoplasmic model.
2. All tiles classified as tumour is step 1 are passed through the object classifier network, Yolov5 for the nuclear model or the membranous model and Mask RCNN for the cytoplasmic model. The classifier network outputs a list of objects with labels per tile. We only consider the objects with labels ‘positive’ or ‘negative’, as these represent tumour cells with either positive IHC staining (‘positive’) or not (‘negative’).
3. The number of positive and negative objects are summed over all tiles. The final score for the WSI is found using:

$score= \frac{num(positive)}{num\left( positive \right) + num(negative)}$

**Supplementary tables**

| Supplementary Table 1. Clinicopathological data for the Gloucester colon cancer stage II cohort | | |
| --- | --- | --- |
| Variables |  | Numbers (%) |
| Number of cases | | 262 |
| Follow-up (Years, Median (Inter quartile range) | | 7.3 (1.9-13.4) |
| Age at surgery, years | | 71.2 (Mean) |
|  | ≤72 | 130 (49.6) |
|  | >72 | 132 (50.4) |
| Sex | |  |
|  | Male | 138 (52.7) |
|  | Female | 124 (47.3) |
| Histological grade | |  |
|  | Well | 54 (20.6) |
|  | Moderate | 155 (59.2) |
|  | Poor | 53 (20.2) |
| Pathological tumour (T) stage | |  |
|  | T3 | 154 (58.8) |
|  | T4 | 108 (41.2) |
| Histological type | |  |
|  | Adenocarcinoma | 232 (88.5) |
|  | Mucinous carcinoma | 29 (11.1) |
|  | Signet ring cell carcinoma | 1 (0.004) |

| Supplementary Table 2. Clinicopathological data for prostate cancer cohort 1 and 2 | | | | |
| --- | --- | --- | --- | --- |
| Variables | | Cohort 1 PTEN* and CCNB1  Number (%) | Cohort 1 Ki-67, β-catenin, CD44, Flotillin1 and Mapre2  Number (%) | Cohort 2, PTEN - validation  Number (%) |
| Number of cases | | 253 | 266 | 259 |
| Follow-up (Years, Median (Inter quartile range) | | 8.2 (5.3-13.2) | 8.2 (5.5-13.0) | 8.5 (4.0-10.2) |
| Age at surgery, years - Mean | | 62 | 62 | 62 |
| Preoperative PSA | | |  |  |
|  | ≤6 | 57 (22.5) | 66 (24.8) | 54 (20.8) |
|  | >6 and ≥10 | 48 (19.0) | 55 (20.7) | 123 (47.5) |
|  | >10 and ≥20 | 84 (33.2) | 79 (29.7) | 73 (28.2) |
|  | >20 | 62 (24.5) | 63 (23.7) | 8 (3.1) |
|  | Missing | 2 (0.8) | 3 (1.1) | 1 (0.4) |
| Extracapsular extension | | |  |  |
|  | Absent | 55 (21.7) | 64 (24.1) | 166 (64.1) |
|  | Present | 196 (77.5) | 220 (82.7) | 89 (34.4) |
|  | Missing | 2 (0.8) | 2 (0.7) | 4 (1.5) |
| Gleason grade group | | |  |  |
|  | 1 (GS ≤6) | 11 (4.3) | 13 (4.9) | 3 (1.2) |
|  | 2 (GS 3+4) | 92 (36.4) | 102 (38.3) | 153 (59.1) |
|  | 3 (GS 4+3) | 77 (30.4) | 76 (28.6) | 54 (20.8) |
|  | 4 (GS 8) | 44 (17.4) | 46 (17.3) | 12 (4.6) |
|  | 5 (GS 9-10) | 29 (11.5) | 29 (10.9) | 37 (14.3) |
| Surgical margins | | |  |  |
|  | Negative | 91 (36.0) | 100 (37.6) | 165 (63.7) |
|  | Positive | 162 (64.0) | 166 (62.4) | 92 (35.5) |
|  | Missing | 0 | 0 | 2 (1.0) |
| Seminal vesicle invasion | | |  |  |
|  | Absent | 187 (73.9) | 210 (78.9) | 228 (88.0) |
|  | Present | 66 (26.1) | 56 (21.1) | 30 (11.6) |
|  | Missing | 0 | 0 | 1 (0.4) |
| Lymph node invasion | | |  |  |
|  | Absent | 239 (94.5) | 253 (95.1) | 252 (97.3) |
|  | Present | 14 (5.5) | 13 (4.9) | 7 (2.7) |
| Capra-S risk group | | |  |  |
|  | Low | 30 (11.9) | 35 (13.2) | 100 (38.6) |
|  | Intermediate | 87 (34.4) | 98 (36.6) | 93 (35.9) |
|  | High | 132 (52.2) | 128 (48.1) | 58 (22.4) |
|  | Missing | 4 (1.5) | 5 (1.9) | 8 (3.1) |

Abbreviations: CCNB1 – Cyclin B, GS - Gleason score, PSA – Prostate specific antigen and PTEN - Phosphatase and tensin homolog.

| Supplementary Table 3. Clinicopathological data for the 142 cases in the Oslo 2 (Osl2) cohort | | |
| --- | --- | --- |
| Variables |  | Numbers (%) |
| Number of cases | | 142 |
| Age at surgery, years - Mean | | 57 |
| Histological grade | |  |
|  | 1 | 16 (11.3) |
|  | 2 | 55 (38.7) |
|  | 3 | 55 (38.7) |
|  | Missing | 16 (11.3) |
| Pathological tumour stage | |  |
|  | I | 48 (33.8) |
|  | II | 65 (45.8) |
|  | III | 12 (8.4) |
|  | Missing | 17 (12.0) |
| Histological type | |  |
|  | Intraductal carcinoma, NOS | 108 (76.1) |
|  | Lobular carcinoma in situ | 10 (7.0) |
|  | Other types | 11 (7.7) |
|  | Missing | 13 (9.2) |

Abbreviations: NOS – not othervise specified

Supplementary Table 4. Endometrial cancer

| Supplementary Table 3. Clinicopathological data for the 1228 cases with in the endometrial cohort OUH | | |
| --- | --- | --- |
| Variables |  | Numbers (%) |
| Number of cases | | 1228 |
| Follow-up (Years, Median (Inter quartile range) | | 7.6 (5.0-11.8) |
| Age at surgery, years - Mean | | 68.3 |
| Grade | |  |
|  | 1 | 462 (37.6) |
|  | 2 | 273 (22.2) |
|  | 3 | 155 (12.6) |
|  | Missing | 338 (27.5) |
| Pathological tumour stage | |  |
|  | I | 839 (68.3) |
|  | II | 79 (6.4) |
|  | III | 215 (17.5) |
|  | IV | 95 (7.7) |
| Histological type | |  |
|  | Endometrioid | 880 (71.7) |
|  | Serous | 132 (10.7) |
|  | Clear cell | 35 (2.9) |
|  | Carcinosarcoma | 84 (6.8) |
|  | Mucinous | 7 (0.6) |
|  | Mixed | 67 (5.5) |
|  | Unclassified/Undifferentiated | 23 (1.9) |

Abbreviation. OUH - Oslo University Hospital.

*Supplementary Table 5. List of antibodies and visualization methods*

| **Primary Antibody** | **Supplier** | **Clone/Kit** | **Dilution** | **Visualization** | **Scanner** |
| --- | --- | --- | --- | --- | --- |
| Ki-67 | Agilent | MIB-1 | 1:200 | EnVision FLEX + | Leica, Aperio AT2 |
| PMS2 | Agilent | EP51 | 1:25 | EnVision FLEX + | Hamamatsu, NanoZoomer (NZ) XR |
| MSH6 | Agilent | EP49 | 1:50 | EnVision FLEX + | Hamamatsu, NZ XR |
| PTEN | Cell Signaling Technology | 138G6 | 1:400 | EnVision FLEX + | Hamamatsu, NanoZoomer XR |

| CCNB1 | Abcam | Y106 | 1:200 | EnVision FLEX + | Hamamatsu, NZ XR |
| --- | --- | --- | --- | --- | --- |
| β-catenin | BD Transduction Laboratories | 14 | 1:2500 | EnVision FLEX + | Hamamatsu, NZ XR |
| CD44 | Agilent | DF1485 | 1:50 | EnVision FLEX + | Hamamatsu, NZ XR |
| Mapre2 | LSBios | 1F3 | 1:3250 | EnVision FLEX + | Hamamatsu, NZ XR |
| Flotillin 1 | Atlas antibodies | Polyclonal | 1:700 | EnVision FLEX + | Hamamatsu, NZ XR |
| ER | Clinical setting |  |  |  | Hamamatsu, NZ XR |
| PR | Clinical setting |  |  |  | Hamamatsu, NZ XR |

Abbreviations: CCNB1 – Cyclin B, ER – Estrogen receptor, MSH6 - MutS Homolog 6, PR – Progesterone receptor, PMS2 - PMS1 Homolog 2, Mismatch repair component, , PTEN - Phosphatase and tensin homolog.

Supplementary Table 6. Overview of human experts making manual labelling, manual counts and manual scoring of protein expression in the datasets in the study.

|  | **Protein** | **Material** | **Development** | **Test (Manual counts) Observer 1** | **Test (Manual counts) Observer 2** | **Manual scoring Observer 1** | **Manual scoring**  **Observer 2** |
| --- | --- | --- | --- | --- | --- | --- | --- |
| Development sets | Ki-67 | Colon | 1,7,8 | 1 | 5 | 3 | 1 |
|  | PMS2 | Colon | 1 | 1 | 2 | 2 | 1 |
|  | PTEN | Prostate | 4 | 4 | 2 | 4 | 2 |
|  | β-catenin | Prostate | 1,4 | 1 | 2 | 3 | 1 |
| Validation sets | MSH6 | Colon |  |  |  | 2 | 1 |
|  | PMS2 | Endometrial |  |  |  | 3 |  |
|  | MSH6 | Endometrial |  |  |  | 3 |  |
|  | Ki-67 | Prostate |  |  |  | 2 |  |
|  | CCNB1 | Prostate |  |  |  | 2 |  |
|  | Ki-67 | Breast |  |  |  | 3 |  |
|  | ER | Breast |  |  |  | 3 |  |
|  | PR | Breast |  |  |  | 3 |  |
|  | CD44 | Prostate |  |  |  | 6 |  |
|  | Flotillin 1 | Prostate |  |  |  | 9 |  |
|  | PTEN | Prostate |  |  |  | 4 | 2 |
|  | Mapre2 | Prostate |  |  |  | 4 |  |

Abbreviations: CCNB1 – Cyclin B, ER – Estrogen receptor, MSH6 - MutS Homolog 6, PMS2 - PMS1 Homolog 2, Mismatch repair component , PR – Progesterone receptor and PTEN - Phosphatase end tensin homolog. 1=WK 2=EE, 3=MP, 4=KC, 5=TMR, 6=LV, 7=ML, 8=AL, 9=KART.

Supplementary Table 7. Patients, whole slide images (WSIs), and tiles in development, test and validation set for deep learning

|  | **Protein** | **Material** | **Scanner** | **#Patients** | **#WSIs/Scans*** | **#Tiles** | **Tile size (pixels)** | **um/pixel** | **#WSIs train / tune** | **#Tiles train / tune** |
| --- | --- | --- | --- | --- | --- | --- | --- | --- | --- | --- |
| Development sets | Ki-67 | Colon | Leica Aperio AT2 | 69 | 69 | 308 | 800 x 800 | 0.253 | 51/18 | 228/80 |
|  | PMS2 | Colon | Hamamatsu XR | 23 | 23 | 528 | 800 x 800 | 0.2267 | 17/6 | 404/124 |
|  | PTEN | Prostate | Hamamatsu XR | 34 | 34 | 3060 | 800 x 800 | 0.2267 | 24/10 | 2160/900 |
|  | β-catenin | Prostate | Hamamatsu NZ | 25 | 25 | 292 | 1024 x 1024 | 0.2285 | 19/6 | 230/62 |
| Test sets | Ki-67 | Colon | Leica Aperio AT2 | 25 | 25 | 75 | 1024 x 1024 | 0.253 |  |  |
|  | PMS2 | Colon | Hamamatsu XR | 24 | 24 | 650 | 800 x 800 | 0.2267 |  |  |
|  | PTEN | Prostate | Hamamatsu XR | 25 | 25 | 675 | 800 x 800 | 0.2267 |  |  |
|  | β-catenin | Prostate | Hamamatsu NZ | 25 | 25 | 300 | 1094 x 1094 | 0.2285 |  |  |
| Validation sets | MSH6 | Colon | Hamamatsu XR | 261 | 262 | 618845 | 1024 x 1024 | 0.2267 |  |  |
|  | PMS2 | Endometrial | Hamamatsu NZ | 1297 | 1301 | 2766641 | 1024x1024 | 0.2285 |  |  |
|  | MSH6 | Endometrial | Hamamatsu NZ | 1295 | 1300 | 2681194 | 1024x1024 | 0.2285 |  |  |
|  | Ki-67 | Prostate | Hamamatsu NZ | 266 | 255 | 124281 | 1024 x 1024 | 0.2285 |  |  |
|  | CCNB1 | Prostate | Hamamatsu NZ | 253 | 256 | 123667 | 1024 x 1024 | 0.2285 |  |  |
|  | Ki-67 | Breast | Hamamatsu XR | 129 | 151 | 215888 | 1024 x 1024 | 0.2267 |  |  |
|  | ER | Breast | Hamamatsu XR | 135 | 135 | 274080 | 1024x1024 | 0.2267 |  |  |
|  | PR | Breast | Hamamatsu XR | 142 | 142 | 287255 | 1024x1024 | 0.2267 |  |  |
|  | CD44 | Prostate | Hamamatsu XR | 231 | 268 | 207137 | 1024x1024 | 0.2267 |  |  |
|  | Flotillin 1 | Prostate | Hamamatsu XR | 229 | 229 | 205000 | 1024x1024 | 0.2267 |  |  |
|  | PTEN | Prostate | Hamamatsu XR | 258 | 851 | 1170349 | 800 x 800 | 0.2267 |  |  |
|  | Mapre2 | Prostate | Hamamatsu XR | 217 | 218 | 290949 | 800x800 | 0.2267 |  |  |

*Abbreviations: CCNB1 - Cyclin B, ER - Estrogen receptor, MSH6 - MutS Homolog 6, PMS2 - PMS1 Homolog 2, Mismatch repair component, PR - Progesterone receptor, PTEN - Phosphatase and tensin homolog and WSIs – whole slide images.

Supplementary Table 8. Summary of results obtained by manual scoring and generated by deep learning models in WSIs from the development sets.

| Protein | Expression | | Observer 1 | Observer 2 | Deep learning |
| --- | --- | --- | --- | --- | --- |
|  |  |  | N (%) | N (%) | N (%) |
| Ki-67-colon | Nuclear | Low | 84 (32.2) | 84 (32.2) | 70 (26.8) |
|  |  | High | 167 (64.0) | 162 (62.1) | 175 (67.0) |
|  |  | Missing | 10 (3.8) | 15 (5.7) | 16 (6.1) |
| PMS2-colon | Nuclear (positive controls) | Lost | 53 (20.2) | 53 (20.2) | 47 (17.9) |
|  |  | Present | 206 (78.6) | 206 (78.6) | 215 (82.0) |
|  |  | Missing | 3 (1.1) | 3 (1.1) | 0 (0) |
| PTEN-prostate | Cytoplasmic | Low | 74 (29.2) | 66 (26.1) | 65 (25.7) |
|  |  | High | 175 (69.2) | 183 (72.3) | 184 (72.7) |
|  |  | Missing | 4 (1.5) | 4 (1.5) | 4 (1.5) |
| β-catenin-prostate | Membranous | Low | 66 (24.8) | 12 (4.5) | 63 (23.7) |
|  |  | High | 187 (70.3) | 242 (91.0) | 189 (71.1) |
|  |  | Missing | 13 (4.9) | 12 (4.5) | 14 (5.2) |

*Abbreviations: n – number, PMS2 - PMS1 Homolog 2, Mismatch repair component, PTEN - Phosphatase and tensin homolog, WSI – whole slide images.*

Supplementary Table 9. Summary of results obtained by manual scoring and generated by deep learning models in WSIs from the validation sets.

| **Protein** | **Expression** | | **Observer 1** | **Deep Learning** |
| --- | --- | --- | --- | --- |
|  |  |  | **N (%)** | **N (%)** |
| MSH6-colon | Nuclear | Lost | 6 (2.3) | 2 (0.8) |
|  |  | Present | 254 (96.9) | 260 (99.2) |
|  |  | Missing | 2 (0.8) | 0 |
| MSI-colon  (PMS2 and MSH6) | Nuclear | MSI | 58 (22.1) | 49 (18.7) |
|  |  | MSS | 204 (77.9) | 213 (81.3) |
|  |  | Missing | 0 | 0 |
| PMS2-endometrial | Nuclear | Lost | 326 (26.5) | 334 (27.2) |
|  |  | Present | 896 (73.0) | 891 (72.6) |
|  |  | Missing | 6 (0.5) | 3 (0.2) |
| MSH6-endometrial | Nuclear | Lost | 67 (5.5) | 183 (14.9) |
|  |  | Present | 1147 (93.4) | 1042 (84.9) |
|  |  | Missing | 14 (1.1) | 3 (0.2) |
| MSI-endometrial | Nuclear | MSI | 384 (31.3) | 453 (36.9) |
|  |  | MSS | 828 (67.4) | 771 (62.8) |
|  |  | Missing | 16 (1.3) | 3 (0.2) |
| Ki-67-prostate | Nuclear | Low | 186 (69.9) | 191 (71.8) |
|  |  | High | 70 (26.3) | 64 (24.1) |
|  |  | Missing | 10 (3.8) | 11 (4.1) |
| CCNB1-prostate | Nuclear | Low | 127 (50.2) | 166 (75.1) |
|  |  | High | 94 (37.2) | 55 (24.9) |
|  |  | Missing | 32 (12.6) | 32 (12.6) |
| Ki-67-breast | Nuclear | Low | 15 (11.6) | 18 (13) |
|  |  | High | 114 (83.8) | 111 (86) |
| ER-breast | Nuclear | Low | 26 (19.5) | 28 (21.1) |
|  |  | High | 107 (80.5) | 105 (78.9) |
| PR-breast | Nuclear | Low | 42 (29.6) | 42 (29.6) |
|  |  | High | 100 (70.4) | 100 (70.4) |
| PTEN-prostate Cohort 2 | Cytoplasmic | Lost | 50 (19.6) | 47 (18.1) |
|  |  | Present | 205 (80.4) | 212 (81.9) |
|  |  | Missing | 4 (0.2) | 0 |
| Mapre2-prostate | Cytoplasmic | Low | 115 (50.2) | 108 (46.0) |
|  |  | High | 114 (49.8) | 108 (46.0) |
|  |  | Missing | 6 (2.6) | 19 (8.0) |
| CD44-prostate | Membranous | Low | 62 (26.2) | 57 (24.1) |
|  |  | High | 175 (73.8) | 172 (72.6) |
|  |  | Missing | 0 | 8 (3.4) |
| Flotillin1-prostate | Membranous | Low | 120 (57.1) | 105 (50) |
|  |  | High | 89 (42.4) | 105 (50) |
|  |  | Missing | 1 (0.5) | 0 |

*Abbreviations: CCNB1 – Cyclin B, ER – Estrogen receptor, n=number, MSH6 - MutS Homolog 6, , MSI – microsatellite instable, MSS – microsatellite stable, PMS2 - PMS1 Homolog 2, Mismatch repair component, PR - Progesterone receptor, PTEN - Phosphatase and tensin homolog, WSI – whole slide images.*

Supplementary Table 10. Comparison of manual and deep learning scores for different cut-off levels of microsatellite instability scoring determined by combination of PMS2 and MSH6.

|  | |  |  | |  |
| --- | --- | --- | --- | --- | --- |
| Scoring method | Cut-off % | Scoring | Manual scores (n) | | Correct classification rate (%) |
|  |  |  | MSI | MSS |  |
| Deep Learning | 5 | MSI | 49 | 0 | 96.6 |
|  |  | MSS | 9 | 204 |  |
|  | 10 | MSI | 53 | 0 | 98.1 |
|  |  | MSS | 5 | 204 |  |
|  | 15 | MSI | 55 | 1 | 98.5 |
|  |  | MSS | 3 | 203 |  |
|  | 20 | MSI | 58 | 3 | 98.9 |
|  |  | MSS | 0 | 201 |  |

Abbreviations: MSI – Microsatellite instable, MSS – Microsatellite stable

Supplementary Table 11. Log Rank p-values with hazard ratios and 95% confidence intervals for different cut-off levels for the classification for microsatellite instability status determined by combination of PMS2 and MSH6

|  | Method | p-value Log rank (Mantel Cox) | HR (95% CI) |
| --- | --- | --- | --- |
| Manual | Lost and present | 0.039 | 2.062 (1.020-4.167) |
| Deep Learning | MSI <=5%, >5% MSS | 0.086 | 1.888 (0.901-3.956) |
|  | MSI <=10%, >10% MSS | 0.101 | 1.783 (0.883-3.603) |
|  | MSI <=15%, >15% MSS | 0.049 | 1.993 (0.986-4.028) |
|  | MSI <=20%, >20% MSS | 0.053 | 1.920 (0.979-3.765) |

Abbreviations: CI confidence intervals, HR – Hazard ratio, MSI – Microsatellite instable, MSH6 - MutS Homolog 6, MSS – Microsatellite stable and PMS2 - PMS1 Homolog 2, Mismatch repair component.

**Supplementary Figures**


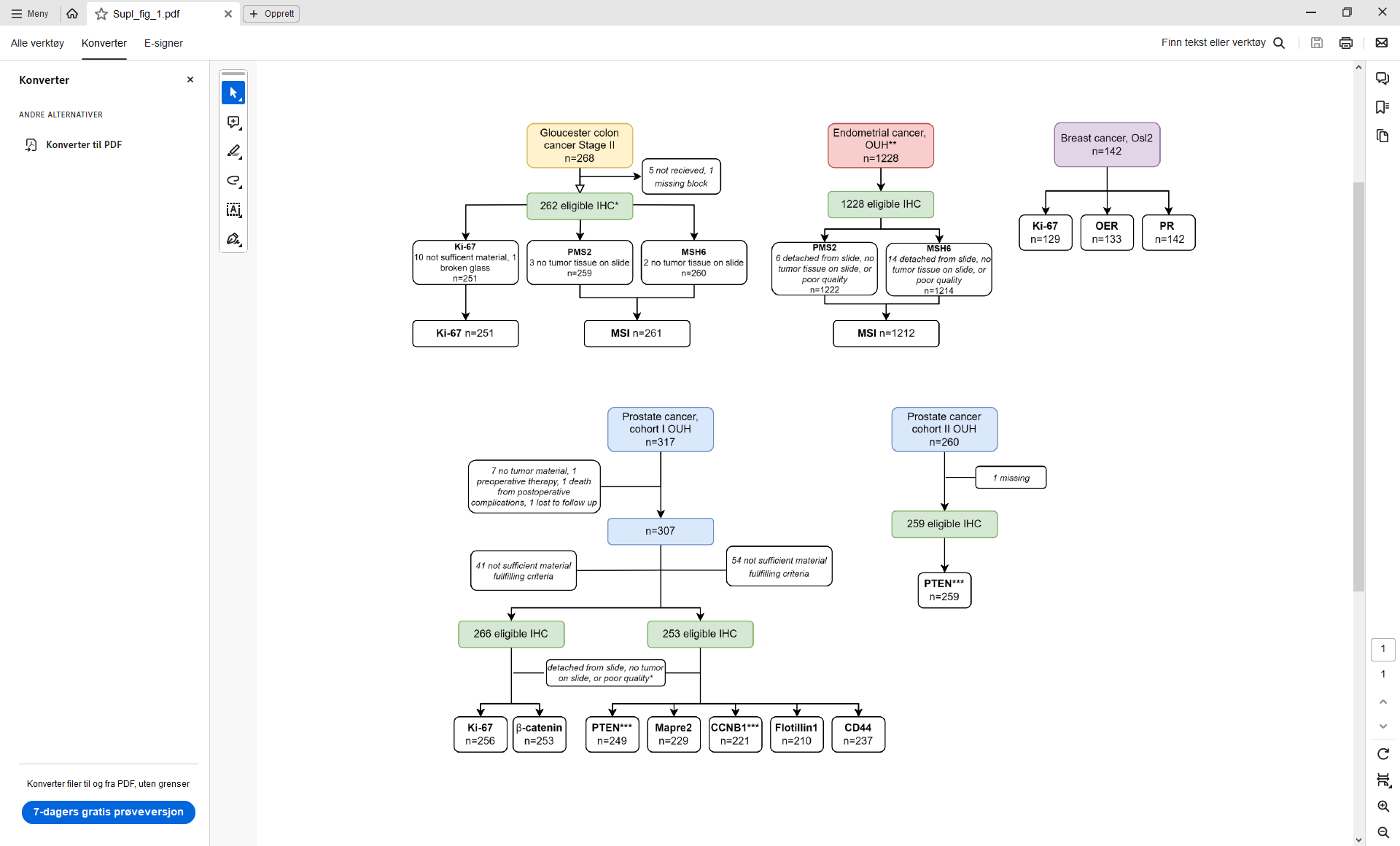


Supplementary Figure 1.


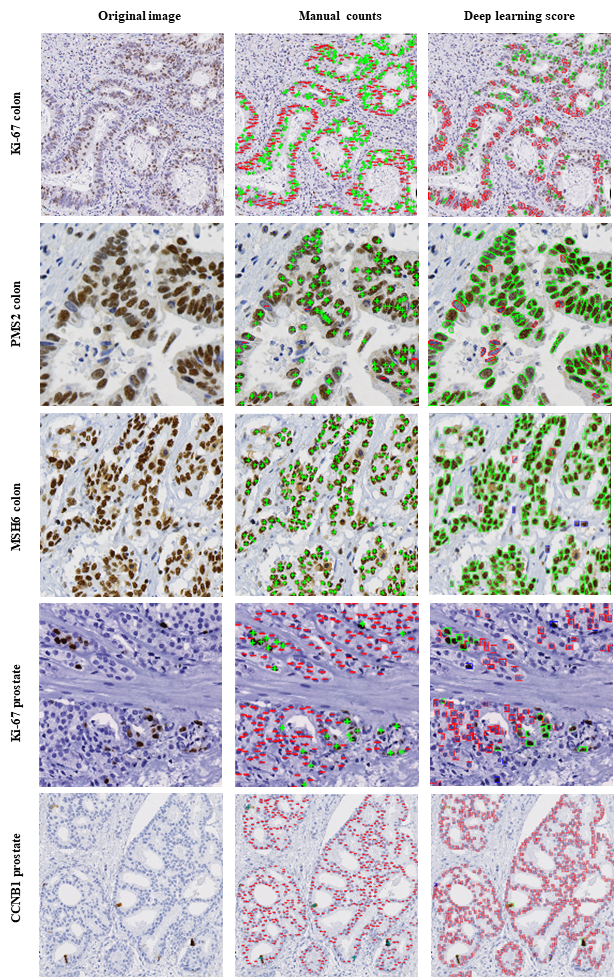


Supplementary Figure 2.1


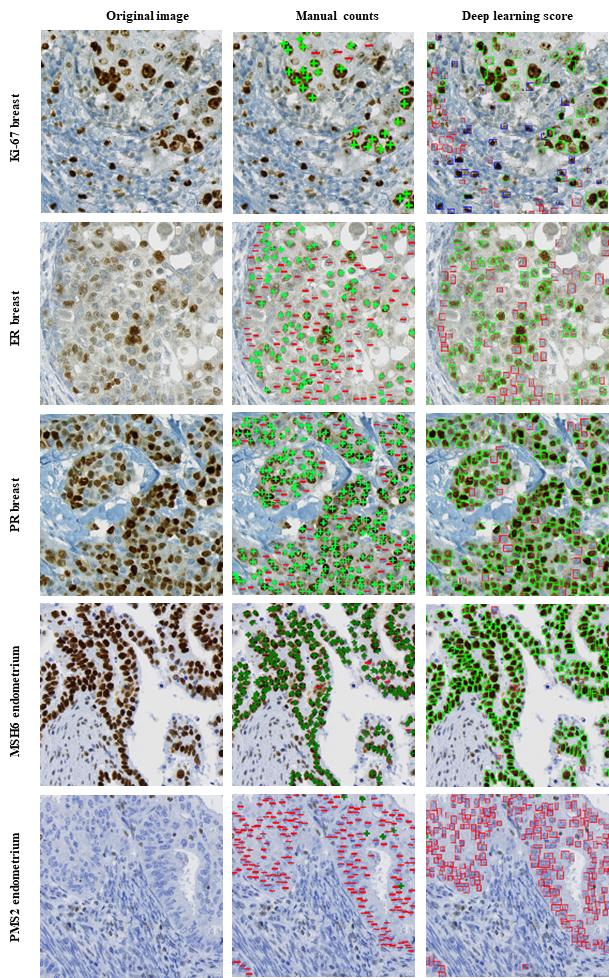


Supplementary Figure 2.2


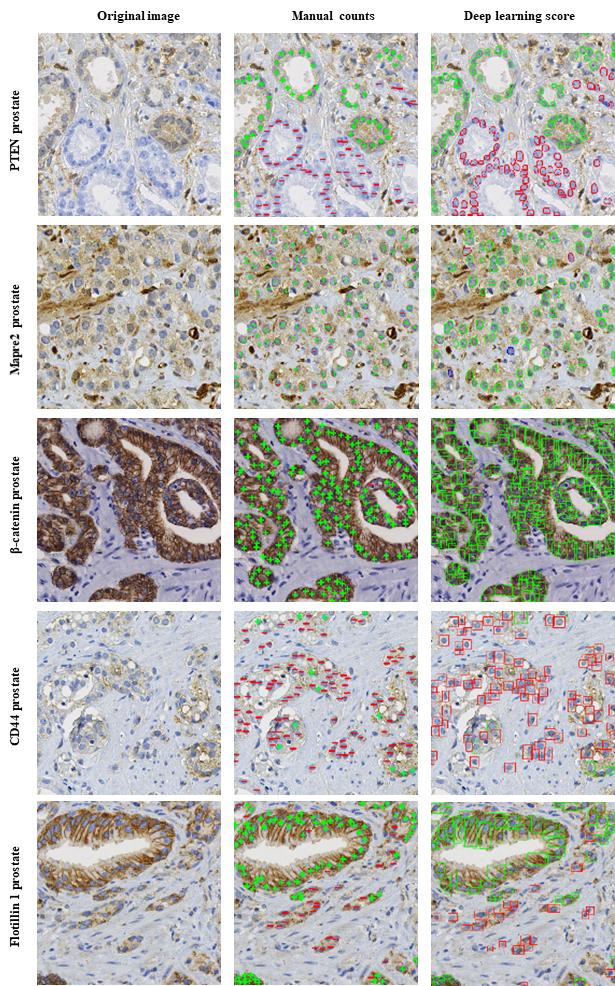


Supplementary Figure 2.3


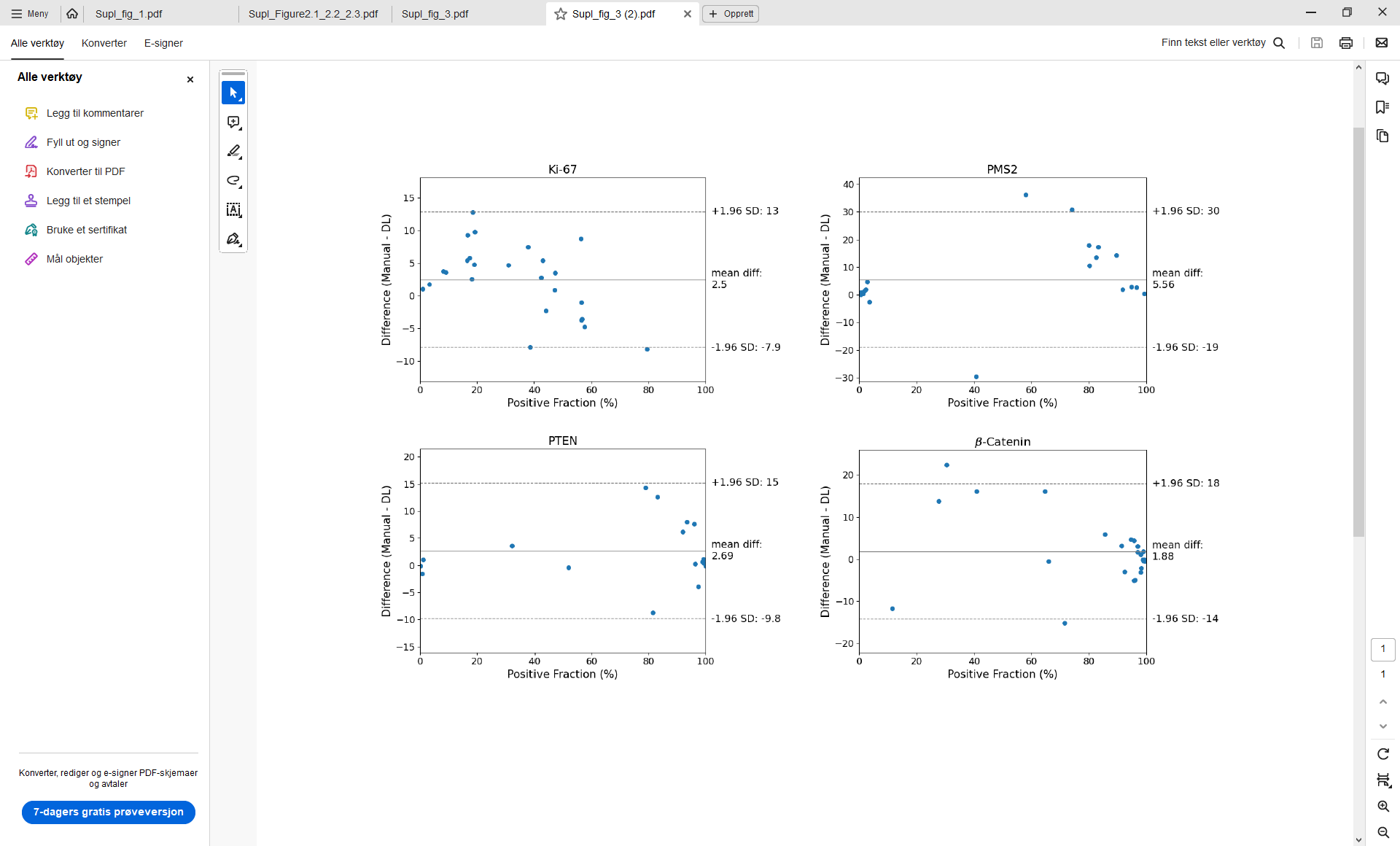


Supplementary Figure 3.

**
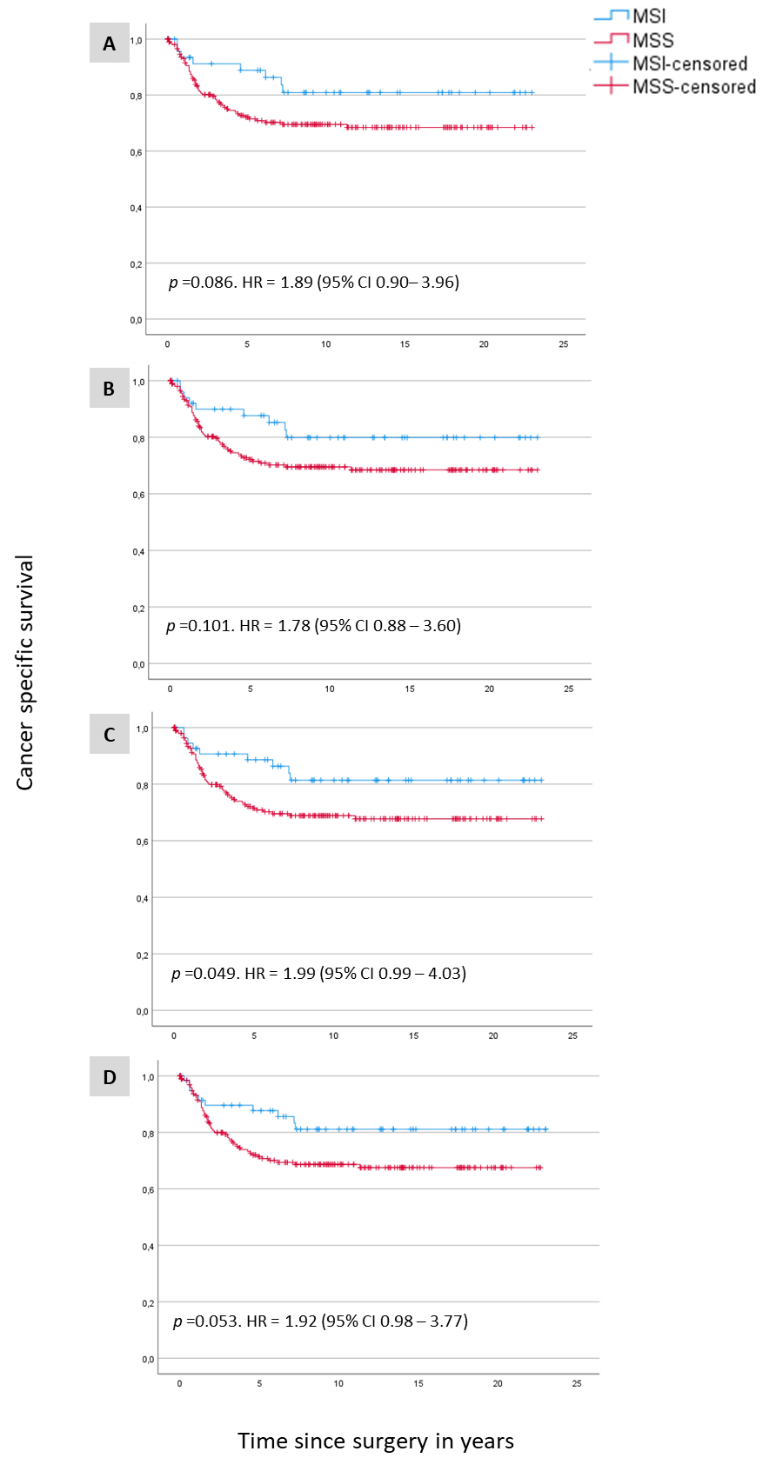
**

Supplementary Figure 4.

**References**

Cyll, K., Kleppe, A., Kalsnes, J., Vlatkovic, L., Pradhan, M., Kildal, W., Tobin, K. A. R., Reine, T. M., Wæhre, H., Brennhovd, B., Askautrud, H. A., Skaaheim Haug, E., Hveem, T. S., & Danielsen, H. E. (2021). PTEN and DNA Ploidy Status by Machine Learning in Prostate Cancer. *Cancers (Basel)*, *13*(17). <https://doi.org/10.3390/cancers13174291>

Redmon, J., Divvala, S., Girshick, R., & Farhadi, A. (2016, 27-30 June 2016). You Only Look Once: Unified, Real-Time Object Detection. 2016 IEEE Conference on Computer Vision and Pattern Recognition (CVPR),

Redmon, J., & Farhadi, A. (2018). YOLOv3: An Incremental Improvement. *ArXiv*, *abs/1804.02767*.
